# Role of Greenland Freshwater Anomaly in the Recent Freshening of the Subpolar North Atlantic

**DOI:** 10.1029/2018JC014686

**Published:** 2019-05-25

**Authors:** D. S. Dukhovskoy, I. Yashayaev, A. Proshutinsky, J. L. Bamber, I. L. Bashmachnikov, E. P. Chassignet, C. M. Lee, A. J. Tedstone

**Affiliations:** ^1^ Center for Ocean‐Atmospheric Prediction Studies Florida State University Tallahassee FL USA; ^2^ Bedford Institute of Oceanography, Fisheries and Oceans Dartmouth Nova Scotia Canada; ^3^ Woods Hole Oceanographic Institution Woods Hole MA USA; ^4^ Bristol Glaciology Centre, School of Geographical Sciences University of Bristol Bristol UK; ^5^ Department of Geographical Sciences Saint Petersburg State University St. Petersburg Russia; ^6^ Nansen International Environmental and Remote Sensing Centre St. Petersburg Russia; ^7^ Applied Physics Laboratory University of Washington Seattle WA USA

**Keywords:** Greenland ice sheet melting, freshwater anomaly, subpolar North Atlantic, subpolar gyre, passive tracer numerical experiment, freshwater budget

## Abstract

The cumulative Greenland freshwater flux anomaly has exceeded 5,000 km^3^ since the 1990s. The volume of this surplus freshwater is expected to cause substantial freshening in the North Atlantic. Analysis of hydrographic observations in the subpolar seas reveals freshening signals in the 2010s. The sources of this freshening are yet to be determined. In this study, the relationship between the surplus Greenland freshwater flux and this freshening is tested by analyzing the propagation of the Greenland freshwater anomaly and its impact on salinity in the subpolar North Atlantic based on observational data and numerical experiments with and without the Greenland runoff. A passive tracer is continuously released during the simulations at freshwater sources along the coast of Greenland to track the Greenland freshwater anomaly. Tracer budget analysis shows that 44% of the volume of the Greenland freshwater anomaly is retained in the subpolar North Atlantic by the end of the simulation. This volume is sufficient to cause strong freshening in the subpolar seas if it stays in the upper 50–100 m. However, in the model the anomaly is mixed down to several hundred meters of the water column resulting in smaller magnitudes of freshening compared to the observations. Therefore, the simulations suggest that the accelerated Greenland melting would not be sufficient to cause the observed freshening in the subpolar seas and other sources of freshwater have contributed to the freshening. Impacts on salinity in the subpolar seas of the freshwater transport through Fram Strait and precipitation are discussed.

## Key Points

- Freshening propagating eastward across the subpolar North Atlantic in the 2010s followed the salinity increase observed since the mid‐1990s
- Simulated content of Greenland freshwater anomaly is high in the western part and low in the Nordic Seas in agreement with observations
- Greenland freshwater anomaly is vertically mixed, leading to small‐salinity decrease suggesting contribution from other freshwater sources

## 1 Introduction

Increased freshening of the subpolar North Atlantic (SPNA) is predicted under present climate conditions due to the amplified freshwater flux from glaciers in the Arctic and subarctic regions, with a consequent impact on convection driving thermohaline circulation (Fichefet et al., 2003; Rahmstorf, 1995; Stouffer et al., 2006; Thornalley et al., 2018; Wu et al., 2004). Greenland freshwater flux has increased by about 200 km^3^/year since 1992 (Bamber et al., 2012, 2018) and, despite a relatively small flux rate, the Greenland freshwater flux anomaly has been persistently positive during that time. The cumulative total freshwater flux anomaly from the Greenland ice sheet integrated over the time period 1993–2016 with respect to the 1958–1992 mean is about 5,000 km^3^. This volume of freshwater has exceeded half of the freshwater volume advected to the North Atlantic from the Arctic Ocean during the Great Salinity Anomaly (GSA) that propagated across the region between the late 1960s and early 1970s (Dickson et al., 1988).

Changes in the ocean salinity and water column stability caused by the Greenland freshwater anomaly (GFWA) have been the central idea in many studies investigating current and near‐future climate change driven by accelerated melting of the Greenland ice sheet (e.g., Fichefet et al., 2003; Proshutinsky et al., 2015; Rahmstorf et al., 2015; Weijer et al., 2012; Yang et al., 2016). For example, Proshutinsky et al. (2015) hypothesized that this surplus freshwater flux played a crucial role in cessation of decadal variability of the Arctic climate. Others have suggested that freshwater flux anomalies from Greenland are already (Rahmstorf et al., 2015) or might, in the near future (Böning et al., 2016; Swingedouw et al., 2006), affect the strength of the Atlantic meridional overturning circulation (AMOC). Nonetheless, many elements of the suggested chain of processes connecting GFWA and climate variability are uncertain, speculative, or contradict observations. One such process is the propagation of the GFWA to the interior Labrador, Irminger, Greenland, and Iceland Seas leading to cessation of deep convection in the convective sites (Swingedouw et al., 2006). For example, an obvious contradiction is recurrent intensification of deep convection in the Labrador and Irminger Seas observed after 2010 (De Jong & de Steur, 2016; Yashayaev & Loder, 2016, 2017) concurring with the ongoing melting of the Greenland ice sheet. Observed deep convection in the SPNA is apparently in disagreement with the predicted slowing of the AMOC caused by the GFWA.

The uncertainty in the relationship between the GFWA and climate is due, in part, to insufficient knowledge about propagation pathways and the accumulation rate of Greenland freshwater in the subpolar seas. A few studies advocate that the Greenland freshwater is rapidly removed from the SPNA, carried by the boundary currents toward the equator (e.g., Moore et al., 2015). Other studies suggest that freshwater anomalies recirculate within the SPNA region for a long time (e.g., Belkin et al., 1998; Yashayaev et al., 2015). This suggestion is supported by Cuny et al. (2002), who showed that surface drifters released in the subpolar North Atlantic stayed within the subpolar gyre, suggesting that the GFWA could recirculate within the region for several years.

Observational data provide controversial evidence for the relationship between the increased Greenland melting and thermohaline changes in the interior SPNA. The widespread freshening observed in the North Atlantic and Nordic Seas during the 1960s to 1990s (Curry et al., 2003; Dickson et al., 2002) reversed to salinification after the early 1990s, resulting in increased salinity that continued until the late 2000s (Holliday et al., 2008; Yashayaev et al., 2015); this is in contrast to the accelerating Greenland melting, which began in the early 1990s.

**Figure 1:**
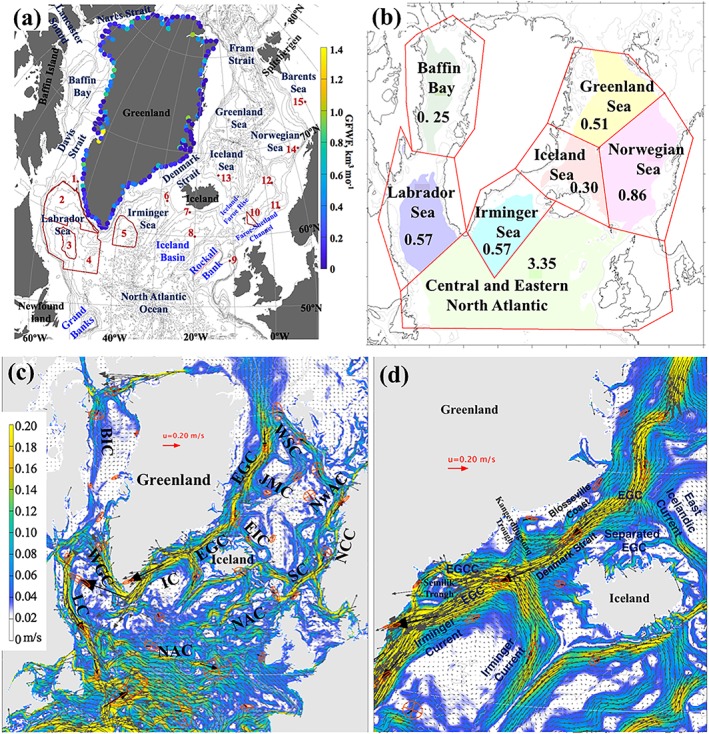
(a) Map of the study region. The isobaths are drawn every 500 m. Colored points along the coast of Greenland show freshwater fluxes (km^3^/month) of the individual freshwater sources in the model forcing field for July 2015 derived from Bamber et al. (2018). The passive tracer is imposed at every location and prescribed tracer flux is proportional to the freshwater flux anomaly. The red numbers designate observational sites where salinity anomalies are analyzed and compared to the model results. 1 = Greenland Fylla Bank, 2 = Northern Labrador Sea, 3 = Central Labrador Sea, 4 = interior deep Labrador Sea, 5 = central Irminger Sea, 6 = northern Irminger Sea, 7 = southwest Iceland shelf, 8 = Iceland Basin, 9 = Rockall Trough, 10 = Faroe‐Shetland Channel, 11 = Southern Norwegian Sea, Svinyo, 12 = OWS “M,” 13 = Iceland Sea, Langanes, 14 = central Norwegian Sea, Gimsoy, 15 = western Barents Sea, Fugloya Bear Island. (b) Selected regions for the tracer and GFWA budget analysis. The color‐shaded regions designate the deep basins inside the 800‐m isobath used in the calculations. The numbers represent the area (10^6^ km^2^) of the shaded regions. (c) Mean ocean surface circulation from the 0.08° Arctic Ocean HYCOM‐CICE. The colors show speed (m/s). The mean vectors and standard deviation ellipses are shown for selected locations. (d) Mean circulation on the eastern Greenland shelf from the 0.08° HYCOM‐CICE resolves major dynamic features discussed in the previous studies (De Steur et al., 2017; Le Bras et al., 2018; Våge et al., 2013). In (c) and (d), abbreviated currents are as follows: EGC = East Greenland Current, EGCC = East Greenland Coastal Current, IC = Irminger Current, WGC = West Greenland Current, BIC = Baffin Island Current, LC = Labrador Current, NAC = North Atlantic Current, SC = Shetland Current, NCC = Norwegian Coastal Current, NwAC = Norwegian Atlantic Current, WSC = West Spitsbergen Current, JMC = Jan Mayen Current, EIC = East Icelandic Current.

The study presented here is motivated by the following research questions: how and at what rate does the GFWA propagate across the SPNA? Where does the GFWA accumulate? And, what is the magnitude and location of salinity changes caused by the GFWA? Answering these questions is critical for understanding the impact of the accelerated Greenland melting on overturning circulation in the region and on the strength of the AMOC. The major goal of this study is to investigate the propagation of the GFWA within the SPNA and quantify the impact of increased Greenland freshwater flux on thermohaline characteristics of the SPNA. The study region includes Baffin Bay, the Labrador Sea, the Irminger Sea, the central and eastern North Atlantic (south of ~40°N), including the Greenland, Iceland, and the Norwegian Seas, collectively known as the Nordic Seas (Figure 1). This paper first analyzes recent hydrographic observations that provide evidence of negative salinity anomalies (freshening) in the SPNA originating in the Labrador Sea and propagating to the eastern North Atlantic and the Norwegian Sea during the time period from the end of 2000s through the 2010s. Then, results from high‐resolution numerical experiments with and without Greenland freshwater flux, and with a continuously released passive tracer along the coast of Greenland at freshwater source locations, are analyzed to assess propagation of the GFWA and related freshwater content (FWC) and salinity change in the region. Lastly, model‐based estimates of freshening are compared with the recently observed freshening in the 2010s to investigate whether the observed salinity changes can be attributed to the accelerating Greenland ice sheet melt. Contributions from the freshwater transport via Fram Strait and precipitation are discussed.

## 2 Data

### 2.1 Greenland Freshwater Flux Data

**Figure 2:**
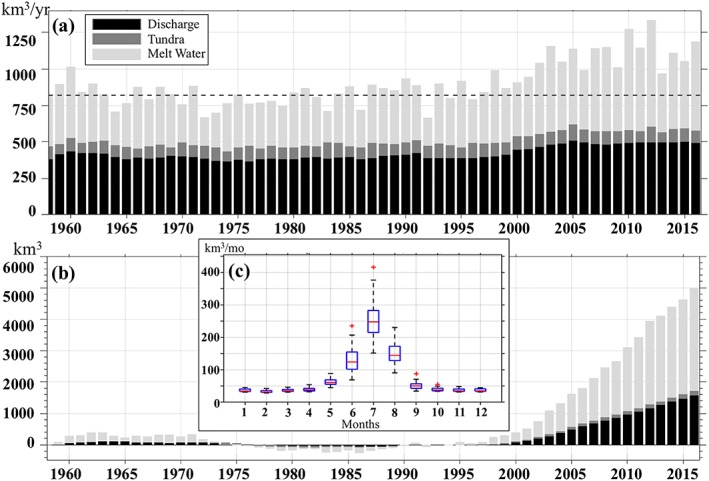
(a) Time series of the annual Greenland freshwater flux (km^3^/year) from Bamber et al. (2018). Stacked bars show freshwater flux components: solid discharge, tundra runoff, and Greenland ice meltwater. The dashed line shows mean total freshwater flux used as a reference to calculate Greenland freshwater flux anomaly. (b) GFWA (km^3^) defined as a time‐integrated Greenland freshwater flux anomaly (equation (3) with t_0_ being 1 January 1959). (c) Box plot diagram of monthly total Greenland freshwater flux (km^3^/month) for 1993–2016.

Locations and discharge rates of the Greenland freshwater sources (Figures 1a and 2) are derived from gridded products described and analyzed in detail by Bamber et al. (2012, 2018). The data set of Bamber et al. (2012) is a gridded product (5 × 5‐km grid) with realistic geographic distribution and temporal variability that provides monthly land ice freshwater flux from the Greenland ice sheet. The updated version (Bamber et al., 2018) also includes freshwater flux from the Arctic glaciers. The Greenland freshwater flux for the model experiments is obtained from the first data set of Bamber et al. (2012) until 2010 and from the second data set of Bamber et al. (2018) for 2011–2016.

### 2.2 Hydrographic Data in the SPNA

The time series of annual mean salinity anomalies at 15 locations of the SPNA (Figure 1a) are constructed from the oceanographic ship survey and profiling float observations. The ship survey data from national and international repeat hydrography and monitoring programs (e.g., the World Ocean Circulation Experiment) and specialized experiments were obtained from the National Oceanic and Atmospheric Administration National Centers for Environmental Information (formerly the National Oceanographic Data Center (https://www.nodc.noaa.gov/OC5/SELECT/dbsearch/dbsearch.html) and Atlantic Zone Off‐shelf Monitoring Program of Fisheries and Oceans Canada (http://www.bio.gc.ca/science/monitoring‐monitorage/azomp‐pmzao/azomp‐pmzao‐en.php)). The Argo data were downloaded from http://www.argodatamgt.org/Access‐to‐data/Access‐via‐FTP‐on‐GDAC. The time series of salinity anomalies for 11 locations (sites 1 and 6–15 in Figure 1a) are derived from the data compiled by the members of the Working Group on Oceanic Hydrography of the International Council for the Exploration of the Sea (ICES) and posted at the ICES Report on Ocean Climate website (http://ocean.ices.dk/iroc/). The oceanographic sections repeatedly occupied throughout the North Atlantic over the past 30 years (Kieke & Yashayaev, 2015) allow extending the time period for accurate assessment of salinity anomalies before the Argo years. Information about the data sources used for constructing the salinity anomaly time series is provided in Table 1.

**Table 1:** Salinity Data

Site #	Locations	Time Period	Data Source	Series Provider
1	Greenland, Fylla Bank	1993–2015	Standard station	TISF, Germany
2	Northern Labrador Sea	1993–2018	Ships and Argo	DFO, Canada
3	Central Labrador Sea	1993–2018	Ships and Argo	DFO, Canada
4	Deep Labrador Basin	1993–2018	Ships and Argo	DFO, Canada
5	Central Irminger Sea	1993–2018	Ships and Argo	DFO, Canada
6	Northern Irminger Sea	1993–2017	Standard station	MRI, Iceland
7	Southwest Iceland Shelf	1993–2017	Standard station	MRI, Iceland
8	Iceland Basin	1993–2017	Extended Ellett Line	SAMS, NOC, UK
9	Rockall Trough	1993–2017	Extended Ellett Line	SAMS, NOC, UK
10	Faroe‐Shetland Channel	1993–2017	Standard station, ships	Multiple, see text
11	Southern Norwegian Sea, Svinoy	1993–2017	Svinoy Section	IMR, Norway
12	Central Norwegian Sea, OWS “M”	1993–2015	Standard station	GFI/UiB, Norway
13	Iceland Sea, Langanes	1993–2017	Langanes Section	MRI, Iceland
14	Northern Norwegian Sea, Gimsoy	1993–2017	Gimsoy Section	IMR, Norway
15	Western Barents Sea, Fugloya Bear Island	1993–2017	Fugloya‐B.I. Section	IMR, Norway

**Figure 3:**
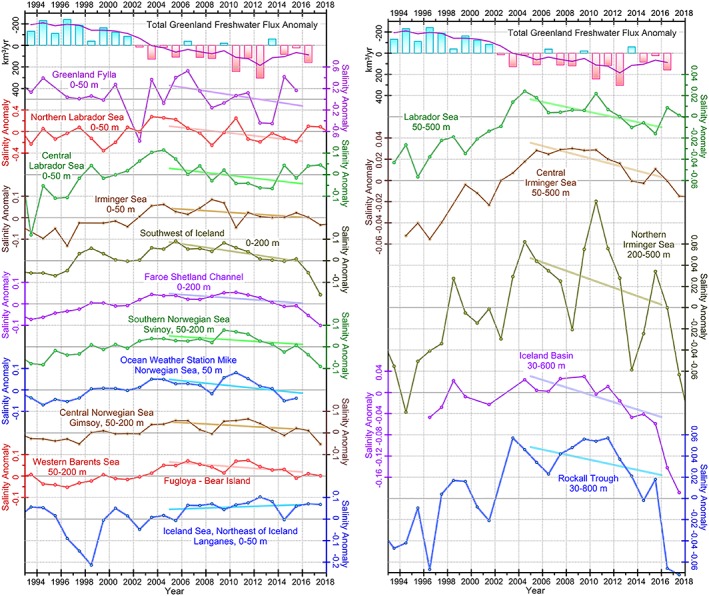
Annual salinity anomalies (circles) in the upper ocean (0–50 and 50–200 m) derived from historical in situ hydrographic observations and Argo floats in the subpolar seas (locations are shown in Figure 1a). The thick light lines are third‐degree polynomial approximating the data. Note different scales for the anomalies. The top bar diagram is the total Greenland freshwater flux anomaly with subtracted mean over 1993–2016. The vertical axis is reversed for ease of comparison with the salinity anomalies. The magenta line is a low‐pass‐filtered time series.

All individual vertical profiles of temperature and salinity observations are quality‐controlled to remove outliers and problematic data (e.g., shifts in Argo float salinity due to conductivity drifts or offsets), low‐pass filtered (with cutoff frequency 1/5 dbar), and interpolated onto a profile with 5‐dbar intervals. A regular seasonal cycle individually estimated at every spatial grid cell (0.5° × 0.5°) is removed from every vertically interpolated salinity profile. The anomalies are then averaged over the chosen layers and later in spatial grid cells by months. These values are used to compute annual anomalies for the regions and locations shown in Figure 1a and compiled in Figure 3. Unlike other sites, the annual salinity anomalies obtained for the Labrador and Irminger Sea polygons are primarily based on year‐round Argo data starting in 2002. In the earlier years, similar to the other chosen sites, only ship data were available with restricted seasonal data coverage.

Salinity anomalies for the shelf and upper slope regions in the northern Labrador and Irminger Seas are derived from salinity time series produced by Thünen Institute of Sea Fisheries and Iceland Marine Research Institute, respectively, and obtained from the ICES website.

For the locations in the eastern North Atlantic and the Nordic Seas (Figure 1a, sites 7–15) the records of annual salinity anomalies downloaded from the ICES Report on Ocean Climate website are used. The Extended Ellett Line mostly occupied by Scottish Association for Marine Science and the National Oceanographic Center (UK) provides salinity anomalies for the sites 8 and 9; a salinity anomaly time series for the Faroe‐Shetland Channel area is obtained for this work by averaging data contributed by the Faroe Marine Research Institute and Marine Scotland Science from three sites in the region indicated on the map (sites 10–12).

### 2.3 Observations in Fram Strait and the Beaufort Gyre

The time series of the monthly mean freshwater transport through Fram Strait are derived from the mooring observations of the Arctic Outflow Observatory (Norwegian Polar Institute) and Alfred Wegener Institute (de De Steur et al., 2018). The transport is calculated relative to a reference salinity of 34.9. The freshwater transport estimates that include the shelf mooring observation are used for this study. These estimates have much higher mean freshwater transport than the time series without the shelf mooring observations (for detail, see De Steur et al., 2018). The time series covers the time period from September 2003 through August 2015. Anomalies of freshwater transport in Fram Strait are calculated relative to the mean transport over 2004–2009, the time period during which the freshwater transport did not exhibit a significant trend (De Steur et al., 2018). Values of FWC in the Beaufort Gyre are estimated from the Beaufort Gyre Observing System hydrographic observations (Proshutinsky et al., 2009). Observations in the Beaufort Gyre cover the time period from 2003 through 2019.

## 3 Model Configuration and Numerical Experiments

### 3.1 The 0.08° Arctic Ocean HYbrid Coordinate Ocean Model–Los Alamos National Laboratory Sea Ice CodE

This study presents numerical experiments conducted with a coupled 0.08° Arctic Ocean HYbrid Coordinate Ocean Model (HYCOM; Bleck, 2002; Chassignet et al., 2006) and Los Alamos National Laboratory Sea Ice CodE (Hunke & Lipscomb, 2008; hereinafter referenced as 0.08° AO HYCOM). The model domain is a subset of the global HYCOM (Metzger et al., 2014) north of ~38°N. A description of the model configuration and computational grid is given by Dukhovskoy et al. (2016). Model characteristics and parameters are summarized in Table 2.

**Table 2:** Parameters of the HYCOM and CICE Models

Model Parameters	Values
HYCOM:	
Horizontal grid	Dipole curvilinear, 0.08
Vertical coordinates	41 hybrid layers
Free surface	Free surface, split time step
Baroclinic time step	180 s
Barotropic time step	3 s
Bathymetry	NRL Digital Bathymetry Data Base 2‐min resolution (DBDB2)
Scalar horizontal advection	Flux‐corrected, second order
Diffusion velocity (m/s) for Laplacian momentum dissipation	−0.00286
Diffusion velocity (m/s) for biharmonic momentum dissipation	−0.02
Diffusion velocity (m/s) for Laplacian thickness diffusivity	0.0
Diffusion velocity (m/s) for biharmonic thickness diffusivity	−0.01
Diffusion velocity (m/s) for Laplacian *T*, *S*, tracer diffusivity	0.005
Quadratic bottom friction	2.2e − 3
Diapycnal diffusivity × buoyancy frequency (m^2^/s^2^)	1.0e − 7
Vertical turbulence	KPP
Maximum viscosity due to shear instability (m^2^/s)	50.0e − 4
Maximum diffusivity due to shear instability (m^2^/s)	50.0e − 4
Background viscosity (m^2^/s)	0.3e − 4
Background diffusivity (m^2^/s)	0.1e − 4
Salt fingering diffusivity factor (m^2^/s)	10.0e − 4
Tracer input	Relaxation to specified concentration values proportional to the freshwater flux at the grid cell
Atmospheric forcing	NCEP CFSR (1993–2010) and CFSv2 (2011–2016)
Lateral open boundaries	1993–2005: 0.08 Global HYCOM+NCODA GOFS3.0 Reanalysis
	2006–2015: 0.08 Global HYCOM+NCODA GOFS3.1 analysis (experiment 53.X)
	2016: 0.08 Global HYCOM+NCODA GOFS3.1 analysis (experiment 53.X)
CICE:	
Ice categories	5
Snow categories	1
HYCOM‐CICE coupling (s)	3600

### 3.2 Improvements to the Model Setup

We have made several improvements to the model setup for the present application compared to the first experiment presented by Dukhovskoy et al. (2016). First, the present model configuration uses improved bathymetry with shallower coastline (5 m instead of 10 m in the old configuration). Then, in the current configuration, HYCOM employs a vertical grid with more vertical layers (41 compared to 32 in the old configuration) that provide higher resolution in the upper 1,500 m. Next, the model experiments are integrated for a longer period of time, from 1993 through 2016, which is the time period that covers the duration of the accelerated Greenland ice sheet melt (Bamber et al., 2012, 2018).

Next, the simulations are driven by continuous atmospheric forcing, Greenland freshwater flux, and more realistic ocean fields imposed at the lateral open boundaries. The 0.08° AO HYCOM is nested within the 0.08° Global HYCOM +NCODA GOFS3.0 reanalysis (Metzger et al., 2014; for 1993–2005) and GOFS3.1 analysis (2006–2016), which provide more realistic ocean fields at the lateral open boundaries. The ocean forcing fields are derived from the daily reanalysis and analysis fields at seven‐day frequency providing realistic lateral fluxes of salt, heat, and mass across the boundaries. No salinity restoring is applied in the experiments. Atmospheric fields are obtained from the National Centers for Environmental Prediction Climate Forecast System Reanalysis (CFSR; Saha et al., 2010) for 1993–2011 and CFSv2 (Saha et al., 2014) for 2012–2016. Finally, the experiment in this study is 10 years longer than the experiment in Dukhovskoy et al. (2016). The duration of the previous model experiment was not long enough to analyze the GFWA pathways in all basins. Some validation results of the 0.08° AO HYCOM are presented in the supporting information. The model performance has been assessed by comparing monthly mean and long‐term mean freshwater fluxes and volume transports through Fram Strait, Davis Strait, Nares Strait, Denmark Strait, and on the southeastern Greenland shelf (Figure S2); computing mean eddy kinetic energy (Figure S3); and calculating February mean mixed layer depth based on Kara et al. (2003) (Figure S4). Evaluation of the 0.08° AO HYCOM shows good agreement between the model and observations (Table S1).

### 3.3 Numerical Experiments

The presented numerical experiments are performed within the Forum for Arctic Modeling and Observational Synthesis project (Proshutinsky et al., 2016). There are twin experiments performed with and without the Greenland freshwater flux imposed along the coast. Both experiments are initialized from a spin‐up simulation started from the ocean climatology fields from the Generalized Digital Environmental Model version 4 (Carnes et al., 2010) and are integrated for 1993–2016. One experiment is conducted without any freshwater flux from Greenland (hereinafter referenced as “GR−”). In the other experiment, the total freshwater flux is imposed at the freshwater sources along the coast of Greenland from the beginning of the simulation (“GR+”). Locations and magnitudes of the Greenland freshwater fluxes (Figure 1a) are prescribed based on Bamber et al. (2012, 2018). Note that in this numerical study, the total Greenland freshwater flux is considered (i.e., the liquid and the solid components are combined) due to the overwhelming complexity of tracking solid ice discharge separately in the employed modeling system.

We analyze the difference in salinity fields simulated in the two experiments in attempt to gauge the impact of Greenland freshwater flux on salinity fields in the subpolar seas. However, the magnitude of this salinity difference fades out quickly away from the coast of Greenland making it difficult to track the spreading of the freshwater in the models. Thus, the propagation and accumulation of Greenland freshwater is also tracked by a passive tracer. In both experiments, the tracer is continuously released along the coast of Greenland at the freshwater sources (Figure 1a), similar to the approach taken by Dukhovskoy et al. (2016). The tracer represents GFWA defined here as the time‐integrated difference between the total Greenland freshwater flux and the mean freshwater flux for 1959–1992 (equation (3)). See Appendix A for details of the tracer implementation in the simulations.

## 4 Observational Data Analysis

### 4.1 Overview of the Greenland Freshwater Flux

The presented study is built upon the first part of the study by Bamber et al. (2018), who analyzed changes in the freshwater fluxes to the North Atlantic caused by accelerated melting of the glaciers. Here a brief overview of the Greenland freshwater flux is provided. The freshwater flux from Greenland is composed of tundra runoff, glacial melt (meltwater), and discharge of solid ice (Figure 2). The Greenland freshwater flux displays a strong seasonal cycle with peak values in July that are 4–6 times higher than the winter mean fluxes (Figure 2c). The contribution of the tundra runoff to the freshwater flux is the smallest (3–5%). The GFWA is the time‐integrated volume of the Greenland freshwater flux anomaly from 1993 to time *t*. The Greenland freshwater flux anomaly is calculated relative to the 1958–1993 mean flux (818.3 km^3^/year or 25.9mSv; the standard error of the mean is 13.2 km^3^/year or 0.42 mSv). The Greenland freshwater flux anomaly shows a nearly steady increase during 1997–2016 (Figure 2b). The largest contribution to this increase is from the meltwater (~65%). The mean Greenland freshwater flux anomaly over the 1993–2016 time period is 211.7 km^3^/year (6.6 mSv); the standard error of the mean is 29.5 km^3^/year (0.9 mSv). Integrated over the time period 1993–2016, the increased freshwater flux from Greenland contributed additional 5,007 ± 390 km^3^ of freshwater (GFWA) to the SPNA.

### 4.2 Salinity Changes in the Subpolar North Atlantic From Hydrographic Data

Hydrographic observations display a notable freshening signal in the upper several hundred meters that spreads over the subpolar North Atlantic between 2005 and 2016 (Figure 3; note the different salinity scales in the diagrams). The time series, except those for the Greenland Fylla site, show a long‐term salinity increase during the 1990s and early 2000s. During 2005–2010, salinity began decreasing at all locations except for the Iceland Sea, which in contrast has experienced an overall salinity increase since 1999. The most pronounced and earliest freshening is observed in the northern Labrador Sea and the Greenland Fylla Bank. The most recent freshening in the Labrador Sea started in 2004–2005. The magnitudes of the salinity anomalies (|δ*S*|) are larger at the northern Labrador Sea (site 2 in Figure 1a) around 0.3 (2009) and 0.2 (2013 and 2015) as compared to the central Labrador Sea where |δ*S*| was <0.1. Further inshore, the series representing the Fylla Bank line southeast of the Davis Strait (“Greenland Fylla,” site 1) shows even stronger freshening signals (|δ*S*| > 0.6) during the 2000s and high magnitude of interannual variability.

The freshening signal weakens as it propagates from the Labrador Sea to the eastern North Atlantic, which is typical for salinity anomalies spreading from the coast of Greenland (e.g., Belkin et al., 1998; Dickson et al., 1988). The salinity anomaly propagates across the upper 500‐m layer of the entire Labrador Sea in 2006–2014 (Figure 3) and continues to spread, with an increasing delay across the Irminger Sea including the northern Irminger Sea (2010–2016), the Iceland Basin (2010–2017), and the Rockall Trough (2011–2017). The anomaly causes a markedly strong freshening in the upper water column in all basins of the subpolar North Atlantic all the way to the Faroe‐Shetland Channel where it arrives around 2011. The freshening of subsurface layers (below the depth of the wind‐driven mixing that is O(100 m)) is related to deep convection driven by extreme cooling associated with strong westerly winds in the winters of 2014 and 2015 (De Jong & de Steur, 2016; Yashayaev & Loder, 2016, 2017). Both of these factors might assist in redistribution of freshwater across the gyre leading to amplification of freshening in the eastern basins. Having passed the Faroe‐Shetland Channel, the low‐salinity anomaly spreads across the eastern Nordic Seas and western Barents Sea. The magnitude of the freshening signal decreases northward. The signal has not yet reached the Iceland Sea, as indicated by the overall slightly positive salinity trend during 2004–2016.

Next, we present results from the numerical experiments to assess if this salinity anomaly observed in the subpolar seas over the last decade might be attributed to the GFWA.

## 5 Model Results

### 5.1 Horizontal Propagation of the Tracer

At every time instance *T* = *t*, the tracer concentration *C*_*tr*_(*t*) provides information about the volume of the GFWA at every location in the model domain D. The local tracer mass that relates tracer to the GFWA volume according to equation (A2) is derived from the simulated *C*_*tr*_ as

(1) $$m_{\mathrm{tr}}\left(x,t\right)=C_{\mathrm{tr}}\left(x,t\right)v\left(x,t\right).$$

**Figure 4:**
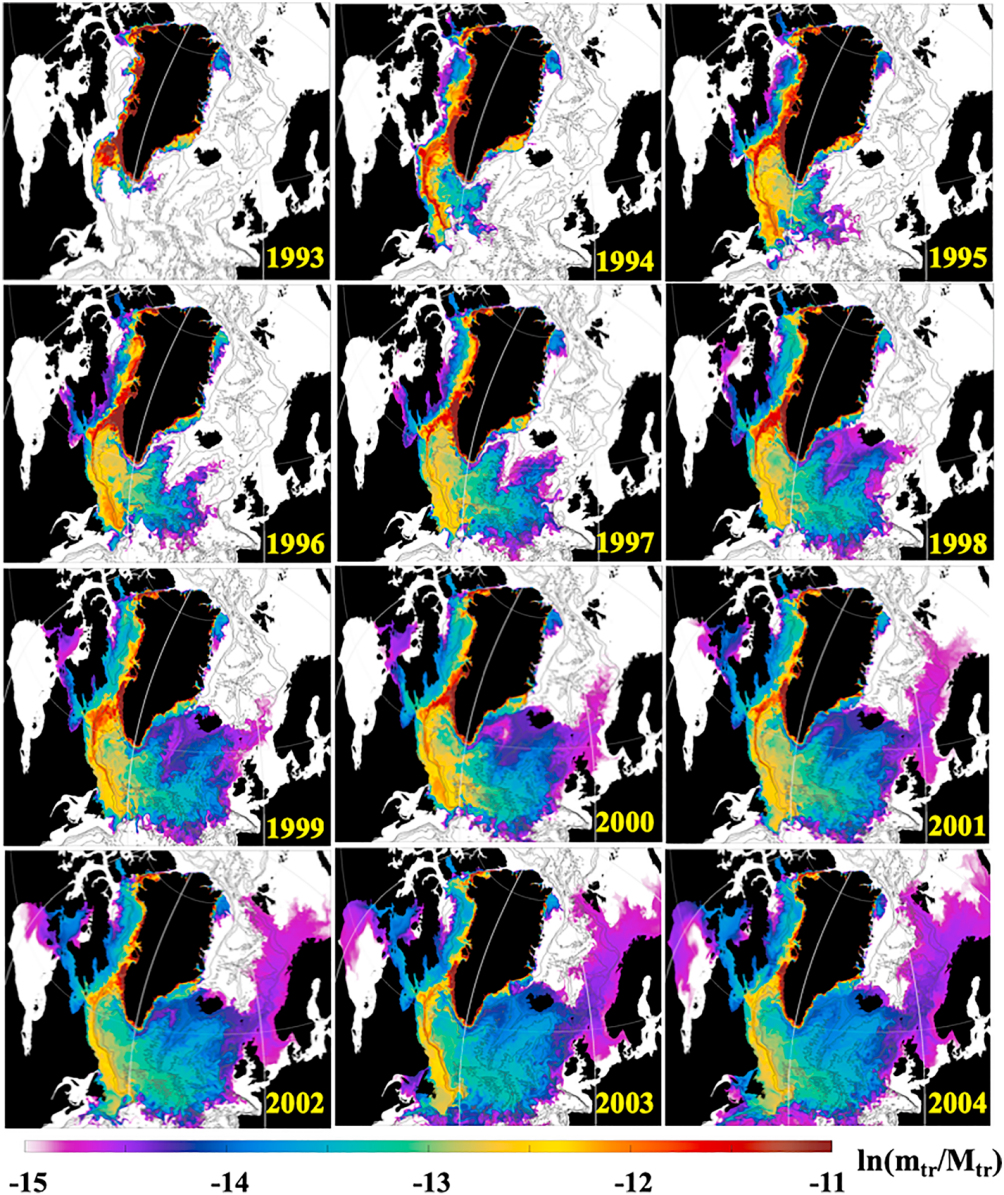
Snapshots of ln (k_Ω_) (equation (2)) derived from the daily mean tracer concentration in the upper 50 m from 0.08° AO HYCOM during 1993–2004. The coefficient is given on a natural logarithmic scale. Each snapshot is for 23 July (for online version, see Animation S5 in the supporting information).

**Figure 5:**
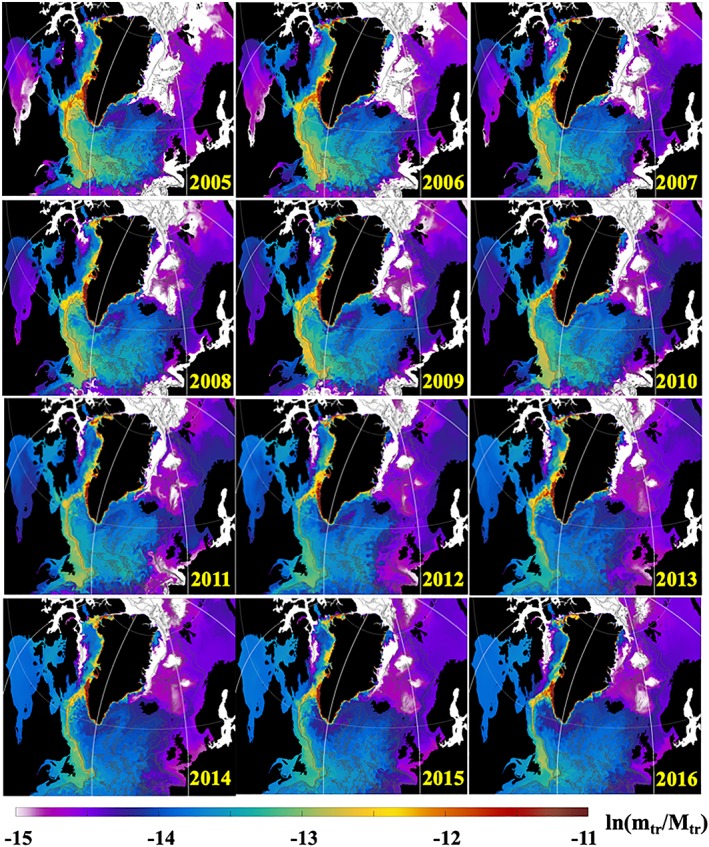
Snapshots of ln (k_Ω_) (equation (2)) derived from the daily mean tracer concentration in the upper 50 m from 0.08° AO HYCOM during 2005–2016. The coefficient is given on a natural logarithmic scale. Each snapshot is for 23 July.

The progression of the Greenland tracer spreading in the SPNA (Figures 4 and 5) is analyzed in terms of a fraction of the Greenland tracer (*k*_Ω_) accumulated within some control volume Ω ⊂ D defined as

(2) $$k_{\Omega }\left(x,t\right)=\frac{\int_{\Omega }C_{\mathrm{tr}}\left(x,t\right)\;dv}{\int_{D}C_{\mathrm{tr}}\left(x,t\right)\;dv}=\frac{m_{\mathrm{tr}}\left(x,t\right)}{M_{\mathrm{tr}}\left(x,t\right)}=\frac{\alpha \;v_{\mathrm{Gr}}}{\alpha \;V_{\mathrm{Gr}}}=\frac{v_{\mathrm{Gr}}}{V_{\mathrm{Gr}}},$$

where *V*_*Gr*_ is the GFWA defined as

(3) $$V_{\mathrm{Gr}}\left(t\right)=\int_{t0}^{t}F_{\mathrm{Gr}}\left(T\right)\mathrm{dT},$$

and *F*_*Gr*_ is the Greenland freshwater flux anomaly (i.e., deviation of the total Greenland freshwater flux from the mean freshwater flux shown with the dashed line in Figure 2a) integrated along the Greenland coast (Figure 1a). Here the control volume Ω is a grid cell bounded by the ocean surface at the top and the geopotential isosurface at some depth.

As follows from equation (2), coefficient *k*_*Ω*_ also reflects the fraction of the GFWA accumulated within the control volume Ω (*v*_*Gr*_) to the total GFWA (*V*_*Gr*_) fluxed into the basin. The presented fields of *k*_Ω_ in Figures 4 and 5 are derived from the daily mean *C*_*tr*_(*t*) on 23 July at every year of the simulation (1993–2016) integrated in the upper 50 m.

The Greenland tracer propagates mainly with the boundary currents (Figure 1c) in agreement with other model studies (e.g., Böning et al., 2016), suggesting that the GFWA generally follows the propagation pathways of the GSA and other salinity anomalies (Belkin, 2004; Belkin et al., 1998; Dickson et al., 1988). However, on the southeastern Greenland shelf, the tracer propagates closely to the coast staying inshore of the East Greenland Current carried by the East Greenland Coastal Current (Bacon et al., 2014; Figure 1d). There is a negligibly small flux of the tracer across the East Greenland Current, which explains the absence of the tracer in the interior Nordic Seas until the tracer is advected into the basin by the North Atlantic current after 12–15 years. After the tracer goes around Cape Farewell, it is transported north with the West Greenland Current. There is remarkable intensification of the tracer lateral advection to the interior Labrador Sea in the southwest Greenland shelf due to the increased eddy activity along the West Greenland Current (Katsman et al., 2004). The pathway of the Greenland tracer splits into two major branches at the northern Labrador Sea. One branch follows the continental shelf break turning southwestward with the Labrador Current. The other smaller branch continues along the west coast of Greenland, advecting the tracer into Baffin Bay. The model simulates tracer accumulation over Fylla Bank. Increased concentration of the tracer in the model suggests an elevated concentration of Greenland freshwater in this region. This result concurs with the fact that during the GSA and other salinity anomaly events—which also propagated around Greenland with the EGC and WGC—freshening had strong manifestation over Fylla Bank (Belkin, 2004; Belkin et al., 1998; Dickson et al., 1988).

Carried by the Labrador Current, the tracer spreads in the Labrador Sea and central North Atlantic. It reaches Newfoundland Island and the Grand Banks within several years, where it mixes with the northward flowing North Atlantic current. Most of the tracer returns into the subpolar basins and some fraction of it flow to the Nordic Seas following the North Atlantic current, in agreement with Cuny et al. (2002). The tracer concentration is more dispersed in the central and eastern North Atlantic. The tracer actively mixes with the ambient waters in the subpolar gyre owing to an increased eddy activity in the region. There is a remarkable difference in terms of the tracer presence in the Nordic Seas and the other subpolar basins. By the end of the simulation (after 24 years), there is less than a third as much tracer in the Nordic Seas as in the subpolar gyre. The interior Greenland and Iceland seas remain the local minima with almost no tracer due to cyclonic circulation in the basins, which averts accumulation of the tracer in the interior regions.

### 5.2 Estimates of the Propagation Time and Accumulation of the GFWA

Simulated tracer concentration is used to evaluate the propagation time of the GFWA within the SPNA. There is some uncertainty in any estimate of the propagation time of a water mass due to the ambiguity of the definition of arrival time and the water mass itself. The arrival time of the water mass is usually defined as a local extremum on a time series of the analyzed characteristic (such as density, salinity or temperature; e.g., Belkin et al., 1998). However, the signal, propagating with the water mass, can be spread over a large area, thus being advected via several pathways. This results in a very broad extremum with several peaks within it. Here the arrival time of the GFWA at a given location (**x**) is estimated as the first instance when *k*_Ω_(**x**,*t*) ≥ 0.8 max (*k*_Ω_(**x,***t*), *t* > 0);equation (2))). This propagation time provides an estimate of an early arrival of the GFWA to a given location and does not correspond to the timing of the maximum content of the anomaly at this location because *V*_*Gr*_ is not constant and increases in time (equation (3)). The estimated propagation time can be interpreted as time required for the incoming and outgoing tracer fluxes to reach a balance within a control volume Ω ⊂ D under steady GFWA flow.

**Figure 6:**
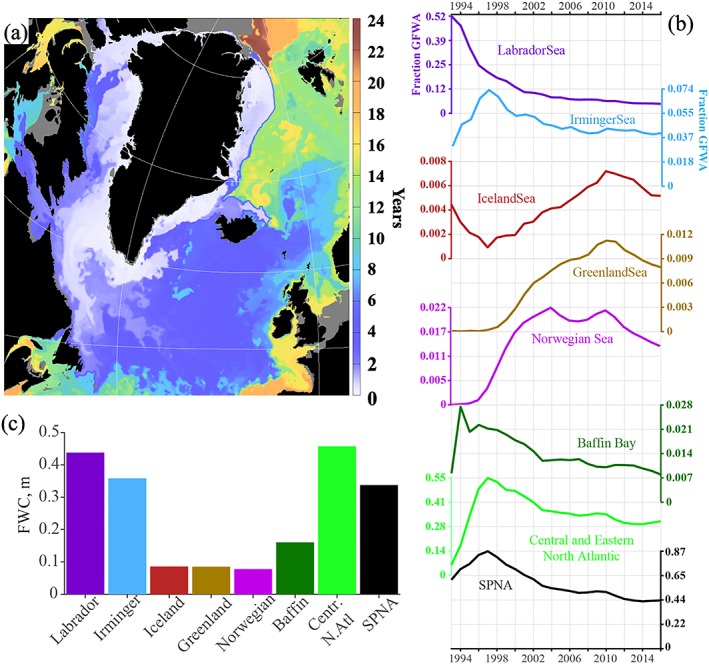
Propagation time and accumulation of the GFWA in the SPNA from the model experiments. (a) Estimated propagation time of the GFWA (years since the beginning of the simulation) from the evolution of the tracer field. The map shows the first instance where k_Ω_(**x**,t) ≥ 0.8 max [k_Ω_(**x,**t), t > 0)]. The grey shading designates regions shallower than 50 m or the near‐zero tracer concentration. (b) Fraction of the GFWA accumulated in the deep subpolar regions (Figure 1b) and overall SPNA. (c) Estimated GFWA content (m) by regions and for the whole SPNA by the end of the simulation.

The SPNA can be divided into several regions based on the estimated propagation time of the GFWA (Figure 6a) into these regions. The GFWA arrives within the first one to two years into eastern and central Baffin Bay and the Labrador Sea. It takes approximately six to seven years for the GFWA to reach the western Baffin Bay. The central and eastern North Atlantic regions are impacted by the GFWA within three to seven years. The anomaly arrives in the southeastern Nordic Seas with the North Atlantic current in approximately six to eight years and spreads over the periphery of the basin during the following four to six years. The interior Greenland and Iceland Seas are impacted by the freshwater anomaly after ~16 years. The distinctly different propagation time of the GFWA to the eastern Greenland shelf (within a year) and adjacent deep basins of the Greenland and Iceland seas (12–14 years) is explained by the very weak shelf‐basin exchange of the Greenland freshwater that tends to flow inshore of the East Greenland Current as discussed in section 5.1 (see also Figure 5).

Accumulation rate of the GFWA in the subpolar basins (defined in Figure 1b) is estimated from the tracer fields using equation (2) where the integration is performed over the whole water depth and Ω being the deep basin bounded by the 800‐m isobath (the shaded areas in Figure 1). Diagrams in Figure 6b show time series of the GFWA fraction within each basin and the whole SPNA. The peaks in the time series correspond to the maximum content of the GFWA in each basin relative to the time‐integrated GFWA (i.e., the ratio of the GFWA in the basin to the total volume of the GFWA released to the North Atlantic). The timing of the peaks agrees with the estimates of the propagation rate of the GFWA shown in Figure 6a. According to the model experiments, ~44% of the cumulative GFWA is retained in the SPNA (~2,200 km^3^) after 24 years. The region including central and eastern North Atlantic is the largest storage of the GFWA (~31%), which also has the largest area compared to the other basins (Figure 1b). The Labrador Sea has the second largest content of the anomaly by the end of the simulation retaining about 5% of the total GFWA. The Irminger Sea has similar to the Labrador Sea storage of the GFWA around 4%. The Norwegian Sea has accumulated ~1.3% of the GFWA by the end of the simulation. The other subpolar basins have less than 1% of the GFWA. The low content of the GFWA in Baffin Bay is counterintuitive; however, it is explained by its smallest area and relatively shallow spreading of the GFWA in the bay (discussed in section 5.4). Accumulated GFWA normalized by the area of an individual basin indicates the Labrador Sea, Irminger Sea, and the central and eastern North Atlantic as the regions of the highest GFWA content (in m per 1 m^2^; Figure 6c).

### 5.3 Greenland Shelf‐Basin Tracer Flux

**Figure 7:**
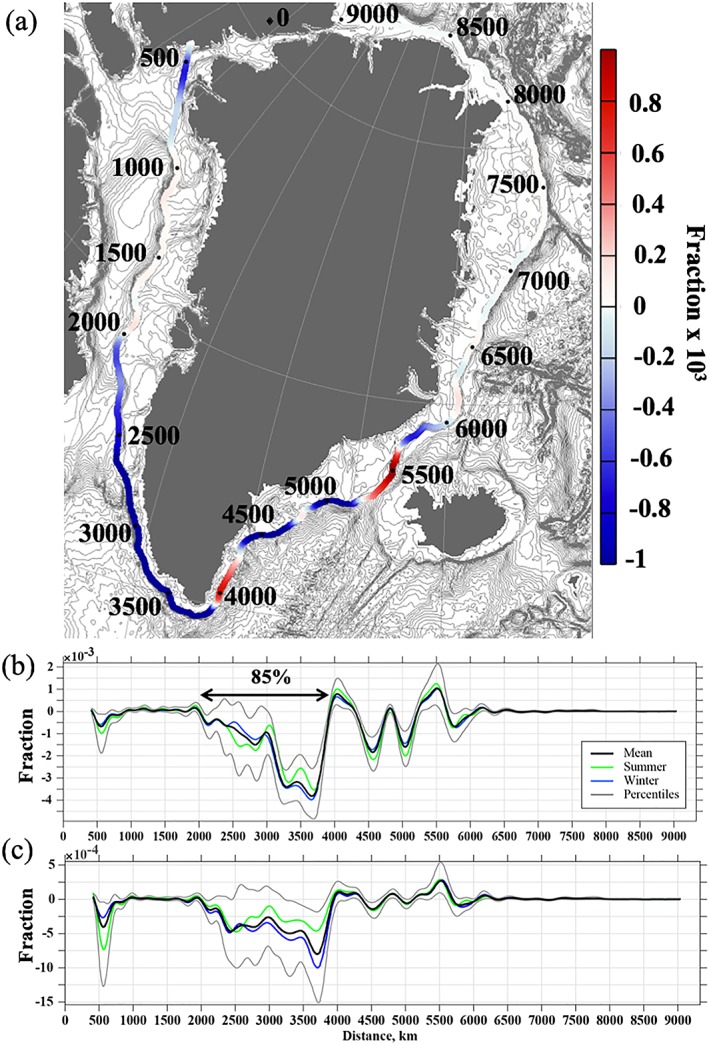
Fraction of the total tracer flux across the contour (equation (4)) per 1 km. Negative flux is directed out of the bounded Greenland domain. (a) The map showing the contour and the flux across the contour. The contour generally follows the 800‐m isobath around Greenland. The numbers on the contour are distances (km). (b) Fraction of the total flux integrated over the whole depth. The arrow depicts the segment of the contour where 85% of the net outflow occurs. (c) Flux integrated over the upper 50 m. In (b) and (c), the blue lines are winter (October–March) and the green lines are summer mean fluxes. The grey lines are the 10th and 90th percentiles.

The importance of the GFWA for climate is its potential impact on deep convection in the SPNA, and resulting impacts on the AMOC. However, the magnitude and location of the freshwater fluxes from the boundary current into the interior convective regions are unknown. The objectives of the following analysis are to identify the regions of the Greenland freshwater shelf‐basin exchange and to analyze the vertical distribution of the tracer in the shelf‐basin fluxes. In order to analyze the shelf‐basin freshwater fluxes, the tracer flux is calculated across a closed contour (∂**Ω**) around Greenland that generally follows the 800‐m isobath (Figure 7a). The total net flux across the contour is

(4) $$F\left(t\right)=\int_{D_{b}\left(x\right)}^{0}\oint_{\partial \Omega }C_{\mathrm{tr}}\left(x,t\right)u\left(x,t\right)\cdot n\;\mathrm{dl}\;\mathrm{dz},$$

where *D*_*b*_ is the local bottom depth, *C*_*tr*_ is the tracer concentration (kg/m^3^), and **n** is a normal vector directed toward Greenland.

The mean Greenland tracer flux is normalized by the total net flux (equation (4)) yielding the fraction of the total depth‐integrated tracer flux along the contour (Figure 7). As expected, the mean net flux for 2000–2016 is strongly negative (i.e., the tracer is fluxed out of the contour), with the strongest tracer outflow across the southwest and south segments of the contour, in good agreement with results from another tracer study by Luo et al. (2016). The northeastern part of the contour has the smallest magnitude of the tracer flux, suggesting minimal spreading of GFWA across the East Greenland Current to the interior Nordic Seas. The currents rapidly advect the tracer distributed by the individual sources along the eastern and southern coast toward the southwestern Greenland shelf where the major shelf‐basin flux of GFWA occurs. The tracer shelf‐basin flux analysis suggests that 85% of the annual depth‐integrated GFWA from the shelf to the ocean occurs across the southwestern Greenland continental shelf break between the Davis Strait and Cape Farewell (Figure 7b). The seasonal signal is quite small over most of the contour and is more noticeable over the southwestern segment. Seasonality is more strongly pronounced in the fluxes integrated over the upper 50 m (Figure 7c) compared to the whole depth‐integrated fluxes (Figure 7b). In general, the outflow is higher during winter. The interannual variability depicted by the 10th and the 90th percentiles (grey lines in Figures 7b and 7c) has the strongest signals over the southwestern segment of the contour. Again, the near‐surface fluxes exhibit stronger signal than the full depth‐integrated fluxes. The obvious disparity in the amplitude of temporal variability in the near‐surface and full‐depth fluxes suggests that this variability can be primarily driven by wind forcing. To test this idea, a simple regression is fitted to the data

(5) $$Q\left(t\right)=\alpha +\beta u_{10}\left(t\right),$$

where *Q* is the anomaly of the monthly mean tracer flux averaged between 2,000‐ and 3,800‐km segment (Figure 7a) and *u*_10_ is the CFSR and CFSv2 10‐m wind vector projected onto the 800‐m isobath such that negative projection corresponds the upwelling‐favorable wind direction in this region. Monthly climatology is removed from both fluxes and winds prior the analysis. The regression has a good fit to data (*p* value of the overall *F* test is <1 × 10^−12^, α = 0, β = 7.6 × 10^−3^, and 8.4 × 10^−3^ for the 50‐m and full‐depth fluxes, respectively). Compared to the full‐depth fluxes, the relationship between the wind and the flux anomalies is much stronger for the near‐surface fluxes. The regression explains 67% of the variability in the near‐surface fluxes versus only 45% for the full‐depth fluxes.

Inspection of the monthly mean fluxes integrated over the whole depth (Figures 7b and 7c) versus the fluxes integrated over the upper 50 m (Figure 7c) reveals relatively homogeneous vertical distribution of the tracer in the water column. The contribution of the subsurface flux (below 50 m) to the total tracer flux is greater than the contribution of the near‐surface flux, despite the fact that the Greenland tracer is released in the model surface layers. This result suggests that the tracer undergoes intense vertical mixing as it travels across the shelf.

### 5.4 Vertical Spreading of the Tracer

Many numerical experiments impose Greenland freshwater directly at the surface layer or distribute it within the upper few meters of the surface layer. Previous studies suggest that some fraction of the Greenland meltwater stays in the subsurface layers, released at depths by the marine‐terminating glaciers (e.g., Beaird et al., 2015, 2018; Straneo et al., 2011). At most observational sites, the GSA and other freshening events were clearly observed only in the subsurface layers (100–500 m; Belkin et al., 1998). At the Ocean Weather Station “Charlie” in the central subpolar gyre, the GSA was discernable down to 1,000 m (Dickson et al., 1988). The mechanisms and the rate of vertical mixing of Greenland freshwater remain uncertain. There are at least two mechanisms responsible for the intensified vertical mixing of freshwater on the Greenland shelf. One is vertical mixing driven by strong along‐coastal winds dominating the Greenland shelf. The other is buoyancy‐driven convection during the cold season on the western Greenland shelf, as discussed by Marson et al. (2017). Here vertical spreading of the passive tracer on the Greenland shelf and deep ocean is analyzed to demonstrate vertical distribution of the GFWA in the water column simulated in the 0.08° AO HYCOM. An advantage of using HYCOM is that in the deep ocean isopycnal layers prevent spurious diapycnal mixing of scalar fields and tracers. The parameterization of the diapycnal mixing in the model is described in Appendix A.

#### 5.4.1 Tracer Vertical Mixing on the Greenland Shelf

Several previous studies discuss an “Ekman straining” mechanism that impacts the horizontal and vertical scales and mixing of the buoyant coastal currents (e.g., Fong & Geyer, 2001; Sanders & Garvine, 2001). During downwelling favorable winds, the current is confined against the coast becoming narrower and thicker with enhanced vertical salt flux. In contrast, during upwelling favorable winds the current spreads off shore and becomes thinner and even more susceptible to vertical mixing of salt. Analysis of high‐resolution hydrographic and velocity transects on the southeast Greenland shelf demonstrates the sensitivity of the East Greenland Coastal Current structure and strength to the along‐shelf wind stress (Sutherland & Pickart, 2008).

**Figure 8:**
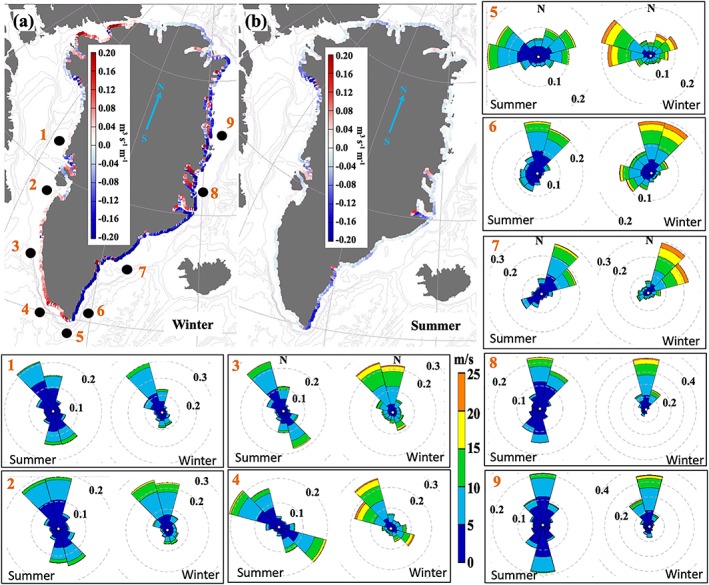
The maps in (a) and (b) show the upwelling index (m^3^/s per 1 m of the coast line) during (a) winter and (b) summer over 1997–2016. The transport is estimated from the CFSR and CFSv2 surface winds. The black dots in (a) and the numbers designate locations for which the wind rose diagrams are presented. In the wind rose diagrams, colors indicate the wind speed (m/s) and the dashed circles are occurrences of winds from the binned directions. Note the difference in orientation of the north in the wind roses and the maps.

In the present study, winter (October–March) and summer (April–September) wind statistics derived from the CFSR and CFSv2 surface wind fields are analyzed for several locations along the coast of Greenland (black bullets in Figure 8a). North winds are the most frequent and strongest over the east Greenland shelf. The central and southern parts of the west Greenland shelf are dominated by north winds in winter, whereas the north and south winds are nearly equal in frequency and strength during summer. North winds are downwelling‐favorable for the east coast of Greenland and upwelling‐favorable for the west coast. North winds push surface water toward the coast impeding the offshore movement of the GFWA (and the tracer). In contrast, north winds over the west Greenland shelf facilitate the offshore spreading of the GFWA (and the tracer) in the Ekman layer. Both of these facts agree with the simulated tracer spreading over the coast of Greenland shown in Figures 4 and 5.

Next, the CFSR and CFSv2 wind fields are used to estimate wind‐driven upwelling and downwelling processes along the coast of Greenland. To do this, the Ekman transport (**M**) is calculated

(6) $$M=\frac{1}{\rho_{w}f}\tau \times k,$$

where *ρ*_*w*_ is the ocean density, *ƒ* is the local Coriolis parameter, and **τ** is the wind stress computed by the bulk formula

(7) $$\tau =\rho_{\mathrm{air}}C_{D}U\left|U\right|,$$

where **U** is the 10‐m wind vector from the reanalysis fields and *ρ*_air_ is the air density. *C*_*D*_ is a drag coefficient computed using the Large and Pond (1981) formulation

(8) CD=10−31.2,U<11ms−10.49+0.065U,11≤U<25ms−12.11525ms−1<U,

The upwelling index (Figures 8a and 8b) is obtained by projecting the Ekman transport vector onto a vector aligned with the bathymetric gradient, positive toward deeper water. Thus, the index is the Ekman transport in the offshore direction (m^3^/s per 1 m of the coast line); i.e., a negative transport corresponds to downwelling.

The locations and seasonality of the upwelling and downwelling events along the coast of Greenland agree with the wind analysis. On average, downwelling events dominate the eastern coast, both in winter and summer, with markedly stronger downwelling events during winter. The southwestern coast is upwelling dominated in winter and downwelling dominated in summer.

Another mechanism that promotes intense deep mixing of the tracer on the western Greenland shelf is convection during the cold season. Numerical studies by Marson et al. (2017) demonstrated formation of dense water on the west Greenland shelf due to brine rejection. Analysis of the vertical mixing on the Greenland shelf from the 0.08° AO HYCOM experiments shows enhanced vertical mixing of the tracer, as well as temperature and salinity, on the west Greenland shelf during winter (not presented), in agreement with Marson et al. (2017).

#### 5.4.2 Analysis of Vertical Propagation of the Tracer in the Deep Ocean

Vertical spreading of the tracer in the North Atlantic by the end of the simulation is assessed from the spatial map of the depth *D*_*R*_, which is the depth of *R* × 100% fraction of the depth‐integrated mass of the tracer at some given location (**x**). The depth is estimated from

(9) $$\frac{\int_{D_{R}\left(x\right)}^{0}C_{\mathrm{tr}}\left(x,z\right)\;\mathrm{dz}}{\int_{D_{b}\left(x\right)}^{0}C_{\mathrm{tr}}\left(x,z\right)\;\mathrm{dz}}=R$$

where *D*_*b*_ is the local total depth and *R* is taken to be 0.8. By the end of the simulation, the deepest values of *D*_*R*_ = 0.8 (1,000–1,500 m) are found in the North Atlantic and the Labrador Sea. About 80% of the tracer is contained within the upper 450–600 m in the interior Baffin Bay. The shoaling of the tracer in the interior Greenland Sea, the Iceland Sea, and the Lofoten and Norwegian Basins is attributed to the mean cyclonic circulation that results in the upward vertical velocities in these regions.

**Figure 9:**
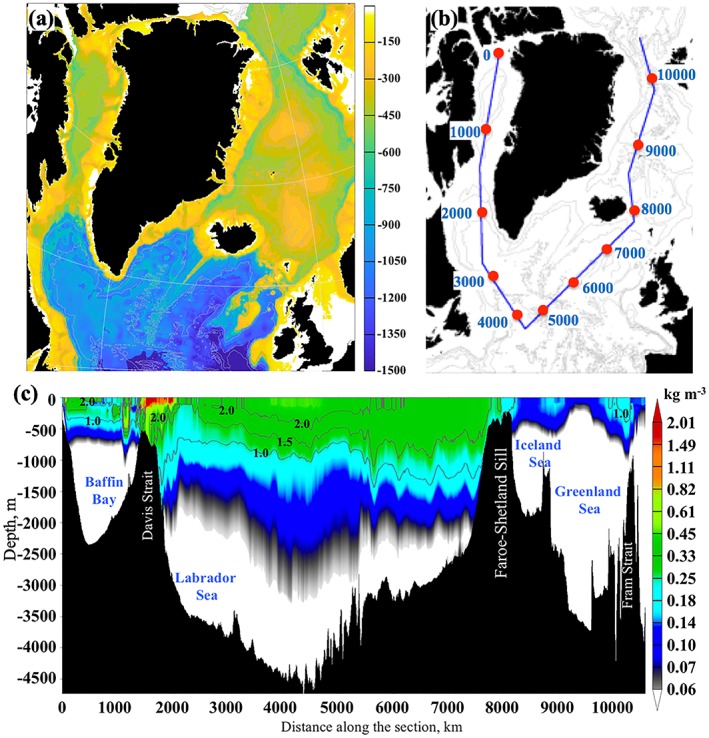
Vertical spreading of the passive tracer. (a) Depth of the layer that contains 80% of the tracer mass in the water column at each location. (b) Section for showing vertical distribution of the tracer concentration (c) with distances (km) along the section. (c) Vertical distribution of the tracer concentration (natural logarithmic scale; kg/m^3^). The isolines show the FWC anomaly (cm of freshwater in 50 m of the water column) caused by the GFWA estimated from the tracer mass budget (see detail in section 4).

Vertical distribution of the tracer by the end of the simulation along the section around Greenland from the Nares Strait to the Fram Strait (Figures 9b and 9c) demonstrates nonuniform vertical spreading of the tracer in different basins. Overall, the highest tracer concentration is maintained in the upper 200 m during the simulation. The concentration is negligibly small, below 500–1,000 m at most locations. For reference, shown in Figure 9c are the contours of FWC anomalies caused by the GFWA accumulated in the water column estimated from the tracer mass budget (*v*_*Gr*_ in equation (2)). The model suggests that the GFWA predominantly stays in the upper 200–500‐m ocean layer. There is substantial accumulation of the GFWA in the Labrador Sea and the North Atlantic that is mixed over 1,000 m, making its signature smeared and less traceable in the real ocean.

Tracer presence in the layers below 100 m in Baffin Bay—below the maximum depth of winter convection in the bay (Tang et al., 2004)—is mainly explained by tracer lateral advection from the shelf (Marson et al., 2017) and the Labrador Sea along the isopycnals. The latter explains the depth of the tracer vertical propagation in the simulation to ~800 m, which is the depth of the isopycnal layer that connects with the Labrador Sea going over the sill in Davis Strait.

## 6 Assessment of Salinity Change Caused by the GFWA

### 6.1 Methodology

Here results of the tracer experiment are used to quantify the contribution of the GFWA to the FWC and expected salinity changes in the SPNA. In order to evaluate salinity change within some control volume Ω ⊂ D caused by the GFWA, the volume of surplus Greenland freshwater accumulated in this volume is needed. According to equation (2), the volume of the GFWA accumulated in Ω at a given time *T* = *t* can be estimated as

(10) $$v_{\mathrm{Gr}}\left(t\right)=k_{\Omega }\left(t\right)V_{\mathrm{Gr}}\left(t\right),$$

where *k*_Ω_ is a fraction of the Greenland tracer accumulated within the volume Ω that is estimated from the simulated tracer concentration field using equation (2). The estimates of *v*_*Gr*_ are then used to calculate FWC and salinity (*S*) changes inside the volume Ω at time *T* = *t*

(11) $${\left.\mathrm{\delta S}\left(x\right)\right|}_{T=t}=-\hat{S}\left(x\right)+\frac{\hat{S}\left(x\right)\left(v_{\Omega }-v_{\mathrm{Gr}}\left(t\right)\right)}{v_{\Omega }},$$

where $v_{\Omega }=\underset{\Omega }{∭}dv$ and $\hat{S}\left(x\right)$ is a reference salinity with respect to which salinity change (*δS*) is estimated. Here $\hat{S}\left(x\right)$ is salinity in 1993 from 0.08° AO HYCOM output fields.

### 6.2 Estimates of Salinity Change From the Tracer Mass Budget

**Figure 10:**
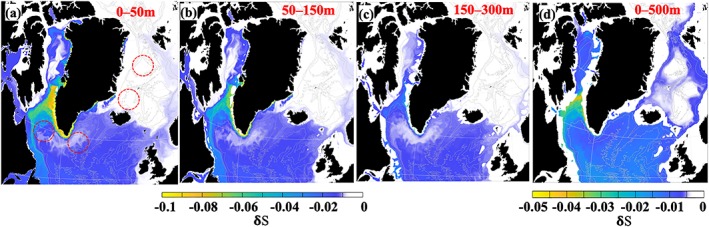
Impact of the GFWA on salinity in the SPNA for layers (a) 0–50 m, (b) 50–150 m, (c) 150–300 m, and integrated over the upper 500 m. In (a), the red circles depict approximate locations of the deep convection regions. Note the different scales for (a)–(c) and (d). Presented results are for the end of the simulation (December 2016).

Changes in salinity within the 0–50‐, 50–150‐, and 150–300‐m layers caused by the GFWA are estimated from the tracer mass budget for December 2016 (the end of the simulation). The volume of the GFWA accumulated in a model grid cell (*v*_*Gr*_) is derived from equation (10), with *V*_*Gr*_ equal to 5,007 km^3^. Then, salinity changes caused by the GFWA at every grid cell are calculated using equation (11). By the end of the simulation, the highest increase in the FWC caused by the GFWA is simulated in the layers within the 0–150‐m depth range on the southwest and southeast shelves of Greenland where salinity dropped by more than 0.1 (Figures 10a and 10b). The north, east, and west peripheries of the Labrador Sea, as well as the shelf in the southern Labrador Sea, have a smaller but notable freshening (0.04 < |δ*S*| < 0.08) in the upper 150 m. Below 150 m, the freshening signal quickly decreases and it is indiscernible below 500 m. The freshening signal in southeastern Labrador Sea and the central Irminger Sea is weak (|δ*S*| < 0.02). In the Nordic Seas, some freshening (|δ*S*|~0.01) occurs in the eastern Norwegian Sea and around the peripheries of the cyclonic gyres in the Greenland and Iceland Seas. The interior Greenland and Iceland Seas (convective sites) are not impacted by the surplus GFW. The depth‐averaged salinity change in the upper 500 m (Figure 10d) is markedly stronger over the Davis Strait sill where the tracer concentration in the water column is high compared to other regions (Figure 9c).

**Figure 11:**
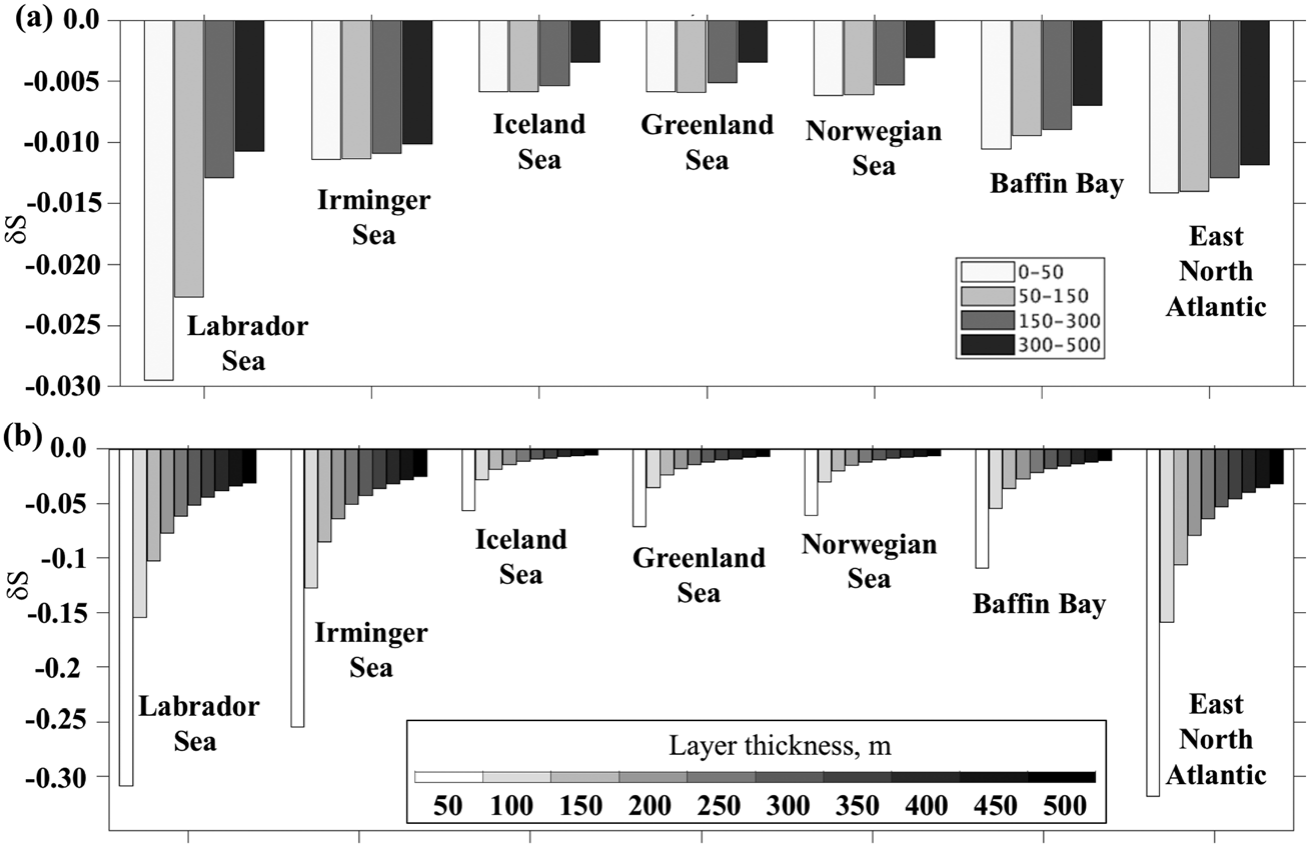
Estimates of salinity changes in the SPNA. (a and b) Estimated salinity changes in the interior regions of the subpolar basins (Figure 1b) caused by the GFWA from the tracer budget analysis for December 2016. (a) Estimated salinity anomaly for 0–50‐, 50–150‐, 150–300‐, and 300–500‐m layers derived from the tracer mass budget. (b) Estimated salinity changes in the layers of varying thicknesses from 50 to 500 m under the assumption that the GFWA is uniformly mixed within the layer.

The estimates of salinity change in the upper ocean depend on the vertical distribution of the GFWA in the water column. The end of the simulation salinity changes in layers caused by the GFWA are averaged over the selected regions (Figure 11a). Then, the depth‐integrated GFWA accumulated in these regions is uniformly distributed within the layers of varying thicknesses (from 50 to 500 m) resulting in salinity anomalies shown in Figure 11b. As expected, the strongest freshening is within the thinnest considered layer (50 m) and decays exponentially as thickness increases. The largest change in the freshening magnitude is in the Labrador and Irminger Seas where the magnitude increases more than 20 times under the most extreme and unrealistic scenario of nearly no mixing, when the tracer would stay in the upper 50 m. Diagrams in Figures 11a and 11b demonstrate that the model simulates a substantial amount of the GFWA accumulating in the water column across the SPNA. Intense vertical mixing results in deep spreading of GFWA preventing it from causing strong freshening in the surface layers.

**Figure 12:**
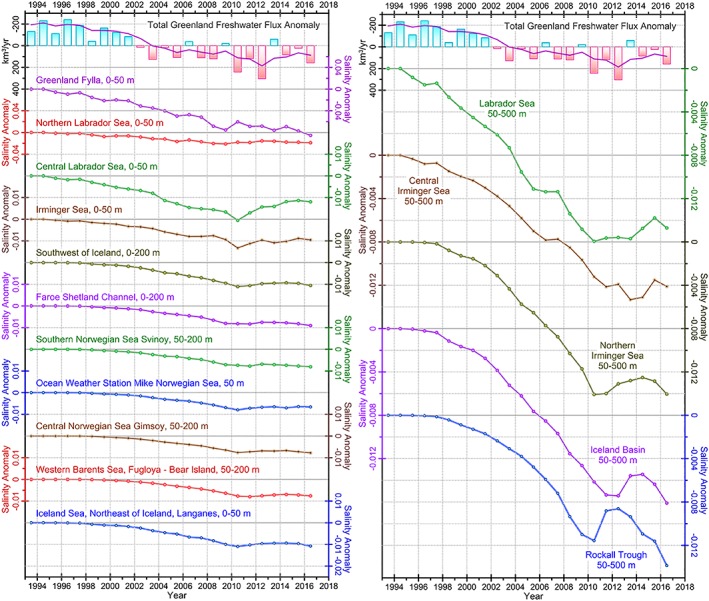
Time series of the salinity change (δS) within the layers 0–50, 50–200, and 50–500 m at the locations shown in Figure 1a caused by the GFWA. The estimates of the salinity change are derived from the tracer budget analysis (equations (9)–(11)). Note different scales.

Time series of salinity changes in the upper ocean layers in the selected regions (at the same locations as the observational sites in Figure 1a) show a gradual decrease of salinity as the GFWA propagates across the domain (Figure 12). The freshening slowly evolves following the increasing Greenland freshwater flux rate and reaches the maximum magnitude after 2008. Simulated freshening is strongest in the upper 150 m of the Labrador Sea and central Irminger Sea where the peak salinity decrease exceeds 0.01. In the eastern North Atlantic the magnitude of salinity change is <0.01. The smallest freshening among the analyzed sites is simulated in the Iceland Sea. As the freshening signal propagates east, it is mixed down into the deep layers revealing more uniform vertical distribution of the salinity anomaly.

### 6.3 Salinity Change From the Experiments with the Greenland Freshwater Flux

Propagation of the freshening signal within the SPNA caused by the accelerated melting of the Greenland ice sheet is assessed by analyzing salinity difference fields from the experiments with (GR+) and without (GR−) the Greenland freshwater flux. Note that here the analyzed changes in salinity are caused by the total Greenland freshwater flux, in contrast to the previous section, which discussed salinity changes caused by the GFWA that are 3–4 times smaller. Therefore, the results presented in this section provide an upper bound of freshening caused by the GFWA and are presented for validation of the salinity change estimates derived from the passive tracer mass budget analyzed in the previous section.

**Figure 13:**
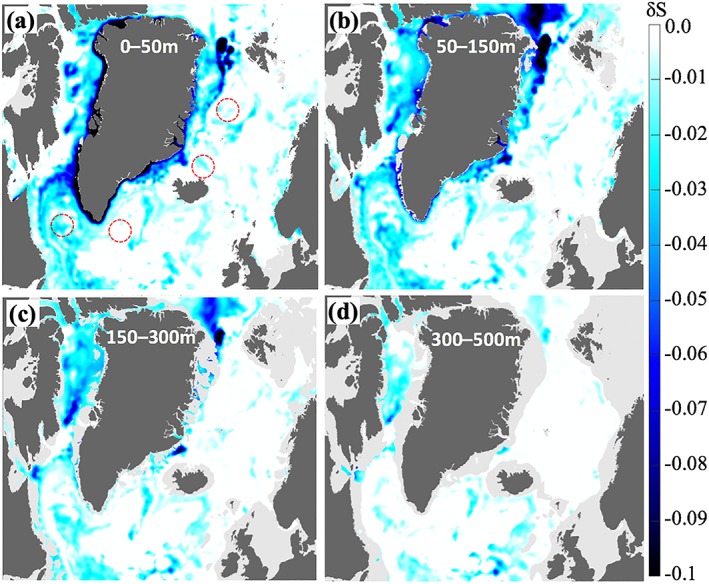
Salinity difference and FWC gain from the GFW experiments. (a–d) Difference maps of the salinity from the simulations with and without Greenland runoff averaged over 2012–2016 in different depth layers. The negative salinity differences indicate lower salinity values in the simulation with the Greenland runoff. The red circles in (b) indicate approximate locations of the deep convection regions.

Mean salinity differences of 2012–2016 from the model experiments demonstrate inhomogeneous and patchy distribution of the freshening signal in the upper 500 m (Figure 13), somewhat contradicting to the tracer‐based estimates (Figure 10), which demonstrate a more uniform spatial distribution of the GFWA within the basin. The reason for this discrepancy in the spatial distribution of the estimated freshening signal is tied to several sources of freshwater (or salt) in the model, including precipitation, melting and freezing of sea ice, freshwater flux from the Arctic Ocean, and imposed salt flux at the lateral open boundary. These sources do not directly impact the tracer, but affect the freshening signal related to the GFWA, making it difficult to track in the model. Different freshwater content in the experiments can also influence ice formation processes and, therefore, the salt flux to the ocean, making the interpretation of the salinity differences between the experiments even more difficult. Additionally, salinity differences in the numerical solutions from the twin experiments result not only from the differences in the imposed Greenland freshwater flux but also from different spatial patterns of mesoscale ocean circulation that evolve due to the nonlinearity of the hydrodynamic system being perturbed at the boundaries.

The values of the tracer‐based salinity change estimates are well within the limits of the freshening magnitude derived from the GR+ experiments. As expected, the total freshwater flux causes stronger freshening in the model with magnitudes exceeding 0.2 near the coast of Greenland and >0.1 in some offshore regions (Figures 13a and 13b) compared to the tracer‐based estimates of freshening caused by the GFWA.

Spatial distribution of the salinity anomaly caused by the Greenland freshwater estimated using two approaches has similar features. The strongest freshening occurs around Greenland, over Fylla Bank, and in the northern Labrador Sea. Both estimates show freshening in the interior Labrador Sea with potential impact on the convective region there. The salinity decrease is stronger in the eastern and interior Baffin Bay in the upper 150 m. Over the domain, the freshening is more pronounced in the upper 300 m and the freshening signal is negligibly small below 500 m. There is strong negative salinity anomaly northeast of Greenland in the 50–150‐m layer that developed in the GR+ simulation. The origin of this anomaly in the simulation is unclear and is being investigated.

## 7 GFWA and Observed Freshening

7

### 7.1 Simulated and Observed Freshening

One of the questions addressed by this investigation is to what extent the observed freshening in the Labrador Sea and eastern North Atlantic of the 2010s is caused by the accelerated Greenland ice sheet melt. The relationship between the GFWA and observed freshening is deduced from the timing and magnitude of the freshening. There is an overall agreement between the timing of the observed salinity decrease (Figure 3) and the evolution of the salinity anomaly estimated from the tracer analysis (Figure 12). The simulated salinity decrease reaches its minimum around 2009–2011 in the Labrador Sea, during 2011–2013 in the Irminger Sea, and around 2010–2016 in the eastern North Atlantic. Around 2008–2010, a freshening begins in the in situ salinity time series matching the timing of the simulated freshening. Note that the modeled freshening begins earlier than that in the observed time series. A possible rationale is that the simulated time series show freshening caused solely by GFWA discarding all other possible processes (such that caused salinification in the SPNA prior to the mid‐2000s) counteracting the surplus freshwater flux and masking its impact.

There are discrepancies between observed and model‐based predicted salinity changes in terms of the magnitudes of salinity anomalies. The freshening estimates derived from the model are smaller than observed salinity anomalies in the SPNA. At the same time, model‐based estimates of the FWC gain caused by the GFWA indicate substantial accumulation of the GFWA within the SPNA (Figures 6b and 6c). The accumulated freshwater anomaly is sufficient to cause freshening with magnitudes comparable to or even higher than the observed salinity change if redistributed over shallower layers (Figure 11b). The model shows, however, that the GFWA is pumped into the subsurface layers in the subpolar gyre. Given that convection to the depths ranging between 1,000 and 2,000 m recurred in the Labrador Sea nearly every year since the start of the record (Yashayaev & Loder, 2016, 2017), the depth of the simulated GFWA mixing in the 0.08° AO HYCOM is realistic. Thus, we conclude that presently the GFWA could not cause the observed freshening in the SPNA during the 2010s. This result agrees with the previous study of Saenko et al. (2017), who also show that the GFWA of similar magnitude (and even double of this magnitude) has negligibly small impact on the SPNA thermohaline fields, barely impacting AMOC. Thus, the present model experiments suggest that additional freshwater sources must have contributed to the recent freshening.

Another conclusion that can be drawn from the model experiments is that the intensity of deep convection in the Labrador and Irminger Seas controls the freshening strength in the SPNA. Weak convection amplifies the freshening signal by distributing GFWA over the thinner layers. In contrast, strong convection disperses the freshwater anomaly over the water column, weakening the manifestation of the freshening signal in the upper ocean. The model results suggest that intensity of the freshwater flux to the SPNA is critical in developing of freshwater anomaly in the near‐surface layers. Slow freshwater fluxes slightly impact water column stability, resulting in deep‐penetrating vertical mixing of the freshwater anomaly. In contrast, the same volume of freshwater fluxed over a shorter period of time would impact the water column stability and weaken deep convection processes, leading to a higher FWC in the near‐surface ocean layers.

### 7.2 Arctic Ocean as a Possible Source of the Freshening in the Subpolar North Atlantic

**Figure 14:**
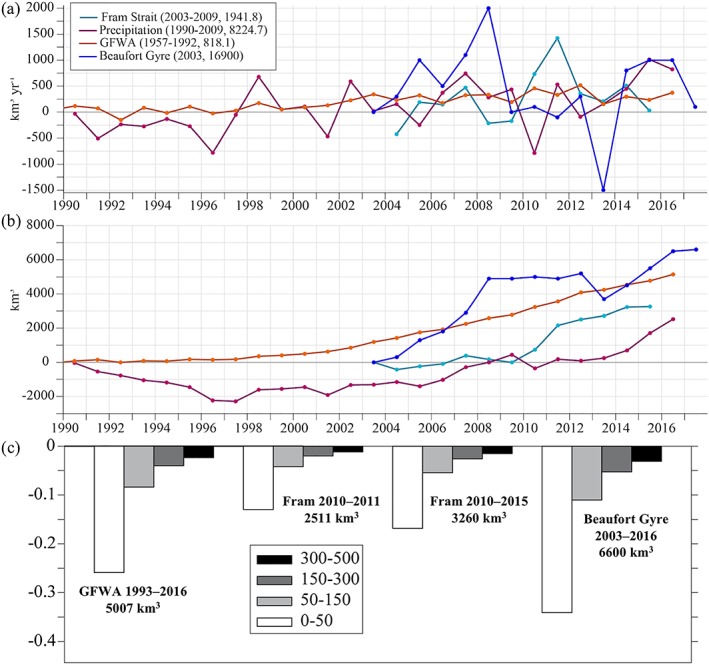
Freshwater flux components in the SPNA and FWC in the Beaufort Gyre. (a) Annual mean anomalies of the freshwater flux components in the SPNA and freshwater accumulation rate in the Beaufort Gyre. Numbers in the legend indicate the averaging period and reference mean flux magnitude (km^3^/year) and initial FWC in the Beaufort Gyre (km^3^). Positive freshwater flux through Fram Strait is southward (toward the SPNA). Annual mean freshwater flux anomaly from precipitation (km^3^/year) integrated over the SPNA. (b) Time‐integrated anomalies of FW flux components and Beaufort Gyre FWC change (km^3^). (c) The bars show salinity change for different layers for the case when the freshwater anomaly from different sources is evenly distributed over the SPNA region. Considered are the GFWA integrated up to 2016, freshwater transport increase in Fram Strait integrated over 2010–2011 and over 2010–2015, the FWC anomaly in the Beaufort Gyre. The numbers on the horizontal axis indicate volume of the freshwater anomalies used to calculate salinity changes in the SPNA.

The most obvious potential source that could have contributed to the freshening that occurred in the 2010s is freshwater outflow from the Arctic Ocean. There are two major routes of Arctic freshwater to the SPNA—through Fram Strait and through the Canadian Arctic Archipelago via Davis Strait. Observations demonstrate increasing FWC of the Arctic Ocean since the late 1990s (Haine et al., 2015; Rabe et al., 2014). Mooring observations from the Beaufort Gyre Observation System (Proshutinsky et al., 2009) show an overall increase of FWC in the Beaufort Gyre since 2003 (Figures 14a and 14b). Importantly, there is no indication of any decrease in the FWC in this region before 2010 that could be attributed to the freshening of the 2010s in the SPNA. Along with the increasing Arctic FWC, no increasing trend in the liquid freshwater export to the SPNA was observed in the Davis Strait and Fram Strait during the 2000s until 2009 (Curry et al., 2014; De Steur et al., 2018; Haine et al., 2015). No significant change in the sea ice volume export through Fram Strait was observed during 2003–2008 (Spreen et al., 2009). Before 2009, one modest increase of liquid freshwater transport from the Arctic Ocean was observed in Fram Strait during 2005–2007 (De Steur et al., 2009; Figure 14a). During this time period, the annual freshwater flux (relative to a salinity of 34.9) increased from ~1,500 to 2,400 km^3^/year. However, because the 2005 freshwater flux had been much lower than the mean freshwater transport of 1,942 km^3^/year (2004–2009), the increase added only 353 km^3^ of surplus freshwater to the North Atlantic (Figure 14b). Although the timing of this increased freshwater transport matches the time of the observed freshening in the Labrador Sea and Fylla Bank (around 2007), the volume of this surplus freshwater flux would be insufficient to cause the freshening of observed magnitude. Since 2009, mooring observations in the Fram Strait (De Steur et al., 2018) reveal a rapid increase of freshwater flux from the Arctic Ocean (Figure 14a). In two years (2010 and 2011), the surplus freshwater export through Fram Strait contributed ~2,150 km^3^ of freshwater to the SPNA totaling 3,260 km^3^ by the end of 2015 (Figure 14b).

Assuming that the mean speed of the East Greenland Current varies within the range from 0.1 to 0.3 m/s (Figures 1c and 1d; also Aagaard & Coachman, 1968; Bersch, 1995), it would take somewhere between 6 and 18 months for freshwater anomaly to travel from Fram Strait to the northern Labrador Sea and four to six years to spread to the eastern North Atlantic. Therefore, the timing of the initial salinity decrease in the Labrador Sea and Fylla Bank (2006–2008) does not match the estimated arrival time of the 2010–2011 Fram Strait freshwater anomaly (around 2010–2011). However, it is possible that the increased freshwater transport through Fram Strait observed during 2010–2011 (and the following years at a smaller rate) could have contributed to the freshening in the SPNA that continued after 2010 (Figure 3). Moreover, the negative trend in some time series of salinity anomalies becomes more negative after 2010.

It is uncertain how strong the freshening caused by this freshwater flux anomaly alone could be. A rough estimate is derived by evenly distributing the volume of the Fram freshwater flux anomaly during 2010–2011 and 2010–2015 over the SPNA uniformly mixed over the layers of varying thicknesses (Figure 14c). Similar estimates from the time integrated GFWA and the Beaufort Gyre FWC anomaly are shown for reference. The depth over which the surplus freshwater is mixed as it spreads over the region is a crucial factor that determines the strength of the freshening signature in terms of salinity change. The estimated salinity change from the increased Fram Strait freshwater flux could be notable if it stays in the upper 150 m. Compared to the similarly estimated freshening caused by the GFWA, the impact of the Fram Strait is smaller. However, according to the 0.08° AO HYCOM results, 44% of GFWA remains in the SPNA. Taking this correction into account, this would make the impact of the Fram Strait freshwater anomaly comparable to or even stronger than the GFWA. It is important to remember that the analyzed GFWA does not include surplus freshwater flux from the glaciers of the Canadian Arctic Archipelago that have contributed an additional ~1,000 km^3^ of freshwater to the SPNA (Bamber et al., 2018). This contribution is comparable to the freshwater anomaly flux in Fram Strait observed in 2006–2008. Assuming the mixing depth of freshwater to be 300–500 m, estimated freshening caused by Fram Strait alone is too small (|δ*S*| < 0.02) to explain observed freshening in the North Atlantic during the 2010s. It is quite possible that both factors (the GFWA and Fram Strait freshwater flux) have combined to cause the development of the recent salinity anomaly.

Another important contributor of freshwater to the SPNA is the Arctic Ocean outflow through the CAA via Davis Strait. Numerical studies using climate models suggest that an increase in the freshwater outflow from the Arctic Ocean to the SPNA will pass via Fram Strait and the CAA (Niederdrenk et al., 2016). Interestingly, accelerated Greenland melt may counteract the freshwater efflux from the Arctic Ocean as proposed by Rudels (2011). Analysis of the Davis Strait moored observations for 2004–2013 did not reveal any significant freshening of the water masses, although negative salinity anomalies were observed in Arctic Water and West Greenland Shelf Water during 2009–2013 (Curry et al., 2014; Dukhovskoy et al., 2016). Unfortunately, the contribution of the CAA freshwater branch is not completely known due to the lack of observations over the last several years. Analysis of the most recent years of (2014–2017) Davis Strait mooring data, currently ongoing, provides an opportunity to identify possible freshening trends in the water flowing to the Labrador Sea.

Air‐sea freshwater flux (precipitation minus evaporation) is another significant factor impacting freshwater content in the SPNA (Josey & Marsh, 2005; Myers et al., 2007). The National Centers for Environmental Prediction/DOE AMIP‐II reanalysis annual mean precipitation rate integrated over the SPNA indicates a possible increase in the air‐sea freshwater flux during the 2000s (Figure 14), assuming an unchanged evaporation rate. A detailed analysis of the air‐sea freshwater flux over the region is needed to investigate the contribution of this freshwater flux to the salinity changes in the North Atlantic.

Looking into the future, the FWC anomaly accumulated in the Beaufort Gyre will likely have a significant impact on thermohaline processes in the SPNA (Figure 14c). There is ~6,600 km^3^ of surplus freshwater accumulated in the Beaufort Gyre that will be released and fluxed to the North Atlantic when the Arctic anticyclonic circulation regime switches to a cyclonic regime (Proshutinsky et al., 2015). The expected impact of this freshwater anomaly on the SPNA salinity will be stronger than that caused by the GFWA merely due to the larger volume (Figure 14c). Compared to the GFWA, it is anticipated that the Beaufort Gyre freshwater anomaly will be released over a shorter period of time and will propagate as a less dispersed water mass causing stronger freshening on the way of its propagation compared to the GFWA. A more accurate investigation of this scenario is needed in order to evaluate possible future changes in the SPNA impacted by a combination of several freshwater anomaly sources.

### 7.3 Other Possible Sources of the Freshening in the Subpolar North Atlantic

Variations in salinity of the North Atlantic current caused by propagating salinity anomalies from the lower latitudes can also impact salinity fields in the SPNA (Curry et al., 2003). For example, decreased salt flux with the North Atlantic current to the SPNA could cause freshening in the region (Glessmer et al., 2014). However, the main effect from decreased salinity of the North Atlantic current would be noticeable in the eastern North Atlantic and the Nordic Seas. On the contrary, observations suggest that the freshening of the 2010s has propagated from the Labrador Sea, leaving GFWA and Fram freshwater outflow the most possible contributors to the freshening. That said, increased salt flux with the North Atlantic current could substantially mitigate the freshening caused by GFWA or Fram Strait. Conversely, decreasing northward salt flux (caused by weakening AMOC; for instance, Smeed et al., 2018) would enhance freshening in the region and promotes weakening of the deep convection (and AMOC), forming a positive feedback mechanism.

## 8 Summary and Conclusions

Analysis of the hydrographic observations in the SPNA reveals freshening originating in the Labrador Sea during 2006–2008 and propagating to the eastern North Atlantic and into the Norwegian Sea. At the same time, there is no indication of the freshening in the Iceland Sea. A possible relationship between the accelerated Greenland melting and the freshening is investigated and tested by numerical experiments. The simulations demonstrate that the GFWA spreads around the SPNA following the boundary currents with substantial lateral mixing into the interior basins, except for the northern part of the EGC. The main outflow of the GFWA to the interior basin occurs at the southwestern part of the shelf. On the northeastern shelf of Greenland, the GFWA propagates close to the coast, staying inshore off the EGC. This prevents the Greenland freshwater from escaping the shelf and results in a very small shelf‐basin exchange with the interior regions of the Nordic Seas. The presence of the GFWA in the Jan Mayen Current and the East Iceland Current is also small in the simulations. Hence, the main route of the GFWA to the Nordic Seas is with the North Atlantic current after the anomaly travels around the Labrador Sea and the southern part of the subpolar gyre.

The timing of the GFWA spreading estimated from the model experiments indicates quick impact (within one to two years) for the western SPNA (Baffin Bay, the Labrador Sea, and the western Irminger Sea). The estimated propagation time of the GFWA to the Nordic Seas exceeds eight years. The convective sites in the Nordic Seas remain nearly unaffected by the GFWA by the end of the simulation (24 years). In contrast, the convective sites in the Labrador and Irminger Seas are impacted by the GFWA.

Tracer budget analysis shows that GFWA is accumulated within the SPNA. By the end of the simulation, ~44% of the GFWA remains in the SPNA. This estimate is higher than the value obtained from the GR+ and GR− experiments (30%), where the GFWA anomaly is calculated from the salinity difference fields. The GFWA is unevenly distributed over the SPNA region with the highest content in the Labrador Sea and the central North Atlantic and the lowest in the interior Nordic Seas.

In the simulations, the GFWA is intensively dispersed vertically in the upper 500 m mainly by winter convection in the Labrador and Irminger Seas, leading to relatively low magnitudes of the freshening in the ocean surface layers. Intense vertical mixing is simulated on the Greenland shelf enhanced by the strong and persistent downwelling favorable north winds over the eastern coast. Winds over the southwestern Greenland shelf are less persistent and alternate between downwelling and upwelling favorable on a seasonal scale. Thus, the GFWA propagates over the interior SPNA as a freshening signal mixed in the upper 300–500 m.

Estimated freshening caused by the GFWA from the simulations suggest persistent negative salinity trend in the upper 300–500 m of the subpolar basins. However, the magnitudes of the estimated freshening are several times smaller than in the observations. The model results suggest that the observed freshening of the 2010s cannot be explained by the GFWA alone and other freshwater sources have contributed to this along with the GFWA. One most possible contributor is freshwater outflow from the Arctic Ocean. The increased freshwater outflow from the Arctic Ocean observed in Fram Strait after 2009 transported 3,260 km^3^ of surplus freshwater that, in combination with the GFWA, could have caused the observed freshening. However, the timing of this freshwater pulse does not explain the origin of the freshening. A more detailed investigation of all freshwater components, including precipitation, and updated results of the observations in Davis Strait are needed to further investigate this problem.

## Supporting information



Supporting Information S1

## Appendix A Implementation of the Passive Tracer in the 0.08° AO HYCOM

Following the implementation of runoff in HYCOM, the tracer and Greenland freshwater flux from a single source are distributed over several ocean grid cells nearest to the freshwater source. The tracer is prescribed in the model by relaxing tracer concentration in the specified ocean grid cells to the maximum concentration value that is set proportional to the local GFWA, as follows. For the given Greenland freshwater flux (*F*_*Gr*_; m^3^/s) at some location (**x**), the tracer concentration (kg/m^3^) is defined as

(A1) $$C_{\mathrm{tr}}\left(x,t\right)=\frac{\alpha \cdot F_{\mathrm{Gr}}\left(x,\;t\right)\cdot t_{\mathrm{rlx}}}{v\left(x,t\right)},$$

where *t*_*rlx*_ is tracer relaxation time scale (one day, here) and *v*(**x**, *t*) is a grid cell volume (m^3^), which is generally a time‐dependent quantity in HYCOM; α is a coefficient of proportionality that relates the tracer mass (*m*_*tr*_) to the volume of GFWA accumulated in a grid cell (*v*_*Gr*_(**x**))

(A2) $$m_{\mathrm{tr}}\left(x,t\right)=\alpha \cdot v_{\mathrm{Gr}}\left(x,t\right).$$

In (A1), α is taken to be 1,000 for creating forcing fields of *C*_*tr*_ for the simulations. The coefficient can be viewed as tracer “density,” as it has units (kg/m^3^). It should be kept in mind that α is merely a proportionality coefficient that can be changed to any value in order to scale the tracer concentration to different values of *F*_*Gr*_ if needed, and it does not have any impact on the tracer advection or diffusion in the model.

A passive tracer is advected and mixed similar to other scalar fields using the same advection‐diffusion equations and turbulence algorithms. In the present experiments, tracers and scalars are advected using a second‐order flux‐corrected transport (Bleck, 1998; Bleck & Benjamin, 1993). Horizontal diffusivity is numerically formulated as

(A3) $$\nu \frac{\partial S}{\partial x}=u_{d}\mathrm{\delta S},$$

where *δS* is a salinity (or tracer) difference between the two adjacent grid cells, and $u_{d}\equiv \frac{\nu }{∆x}$ is a diffusion velocity (Bleck et al., 1992), the value for which is given in Table 2.

In the current simulation, the interior ocean mixing is simulated using the K‐Profile Parameterization (Large et al., 1994) vertical mixing algorithm (Wallcraft et al., 2009). Contributions of resolved shear instability, unresolved shear instability due to the background internal wavefield, and double diffusion are added to the coefficient of the interior diffusivity. Convective overturning is approximated by the K‐Profile Parameterization vertical mixing parameterization by specifying high diffusivity in convectively unstable regions. Under a convective regime, the scalar turbulent velocity scale is estimated based on the convective velocity scale

(A4) $$w^{*}=-{\left(\frac{B_{f}}{h}\right)}^{\frac{1}{3}},$$

where *B*_*f*_ is the surface buoyancy flux and *h* is the surface boundary layer thickness.
