# Supplementary material for: Role of Greenland Freshwater Anomaly in the Recent Freshening of the Subpolar North Atlantic
Source: J Geophys Res Oceans. 2019 May 25;124(5):3333–60. doi: 10.1029/2018JC014686 (PMC6618073; doi:10.1029/2018JC014686)
Supplement: Supplementary file 1 — Supporting Information S1 [file JGRC-124-3333-s001.docx]

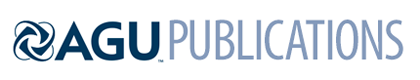


*Journal Geophysical Research - Oceans*

Supporting Information for

**Role of Greenland Freshwater Anomaly in the Recent Freshening of the Subpolar North Atlantic**

Dukhovskoy, D.S., I. Yashayaev, A. Proshutinsky, J.L. Bamber, I.L. Bashmachikov, E. Chassignet, C.M. Lee, A.J. Tedstone

^1^The Florida State University, Tallahassee, Florida, USA.

^2^Bedford Institute of Oceanography, Fisheries and Oceans, Dartmouth, Nova Scotia, Canada.

^3^Woods Hole Oceanographic Institution, Woods Hole, Massachusetts, USA.

^4^University of Bristol, Bristol, UK.

^5^Saint Petersburg State University, St. Petersburg, Russia.

^6^Nansen International Environmental and Remote Sensing Centre, St. Petersburg, Russia.

^7^University of Washington, Seattle, Washington, USA.

**Contents of this file**

Table S1. Freshwater and volume flux estimates in the straits and southeast Greenland shelf from observations

Figure S1. Freshwater and volume fluxes from the 0.08 AO HYCOM-CICE

**Figure S2.** Mean eddy kinetic energy in the upper 50 m from the 0.08 AO HYCOM-CICE simulation and AVISO altimetry.

**Figure S3.** February mean mixed layer depth (m) from the 0.08 AO HYCOM-CICE and subsurface temperature and salinity data from the UK Met Office Hadley Center observations data set version EN.4.1.1 (downloaded fromhttp://www.metoffice.gov.uk/hadobs/en4/on 17 November 2015). The fields are averaged over 2010–2016. The mixed layer depth is estimated using a methodology of (Kara et al., 2003). The model simulates deep mixed layer in the Labradorand Irminger Seas, northeastern North Atlantic in agreement with the EN4 data. Deep mixed layer in the central Greenland Sea is not present in the simulations.

**Movie S1.** Propagation of the passive tracer tracking the Greenland freshwater anomaly. The colors depict a fraction of the Greenland tracer accumulated in the model grid cells in the upper 50 m on a natural logarithmic scale.

**Introduction**

Figures S1–S3 present validation results of the 0.08 AO HYCOM-CICE. The model performance has been assessed by comparing monthly mean and long-term mean freshwater fluxes and volume transports through Fram Strait, Davis Strait, Nares Strait, Denmark Strait and on the southeastern Greenland shelf (Fig. S1); computing mean eddy kinetic energy (Fig. S2); calculating February mean mixed layer depth based on (Kara et al., 2003) (Fig. S3). Evaluation of the 0.08 AO HYCOM-CICE shows good agreement between the model and observations (Table S1). The animation (S1) shows snapshots of spatial distribution of a fraction of the Greenland tracer (*k_Ω_*) in the model grid cells in the upper 50 m. The fraction is derived from the daily mean tracer concentration in the upper 50 m from 0.08 AO HYCOM-CICE during 1993–2016 using Eq. (2). The coefficient is given on a natural logarithmic scale. The animation demonstrates pathways and spreading of the Greenland freshwater anomaly in the SPNA.

**Table S1.** Freshwater and volume flux estimates in the straits and southeast Greenland shelf from observations (negative fluxes are southward)

| Location | Freshwater Flux, mSv (S_ref_) | Volume Flux, Sv | Notes |
| --- | --- | --- | --- |
| Nares Strait | -25 ± 12 (34.8)^(a)^  -31 ± 4 (34.8)^(b)^ | -0.8 ± 0.3^(a)^  -0.9 ± 0.1^(b)^ | Ship observations, early August 2003 |
| Davis Strait | -93 ± 6 (34.8)^(c)^ | -1.6 ± 0.5^(c)^ | Moorings, sea gliders, 2004–2010 |
| Denmark Strait | -65 ± 11 (34.8)^(d)^  -108 ± 24 EGC and -29 ± 7 separated EGC (34.8)^(e)^ | Not measured  Overflow water transport -3.4 ^(e)^ | Mooring observations at the Kogur section, ^(d)^ August 2011–July 2012  ^(e)^ 2008, 2011, 2012 |
| Fram Strait | -63–95 (34.92)^(f)^, -80± 6 (34.92)^(g)^  -70±25 (34.9)^(h)^ | -2± 2.7^(i)^ | ^(f)^Hydrographic observations, Aug-Sep 1997, 1998;  ^(g, i)^ Mooring and ship observations along 79ºN; ^(h)^Moorings including a Greenland shelf mooring along 78.5ºN |
| Southeast Greenland shelf | -74 ± 12 (34.9)^(j)^ | -4.36 ± 0.6 ^(j)^ | Mooring observations, Overturning in the SPNA, 2014–2016 (~60 ºN, east across the shelf). East Greenland Coastal Current + EGC |

^(a)^Münchow et al., 2006

^(b)^Münchow et al., 2007

^(c)^Curry et al., 2014

^(d)^De Steur et al., 2017.

^(e)^Våge et al., 2013.

^(f)^Meredith et al., 2001

^(g)^Rabe et al., 2009;

^(h)^De Steur et al., 2018

^(i)^Schauer et al., 2008

^(j)^Le Bras et al., 2018


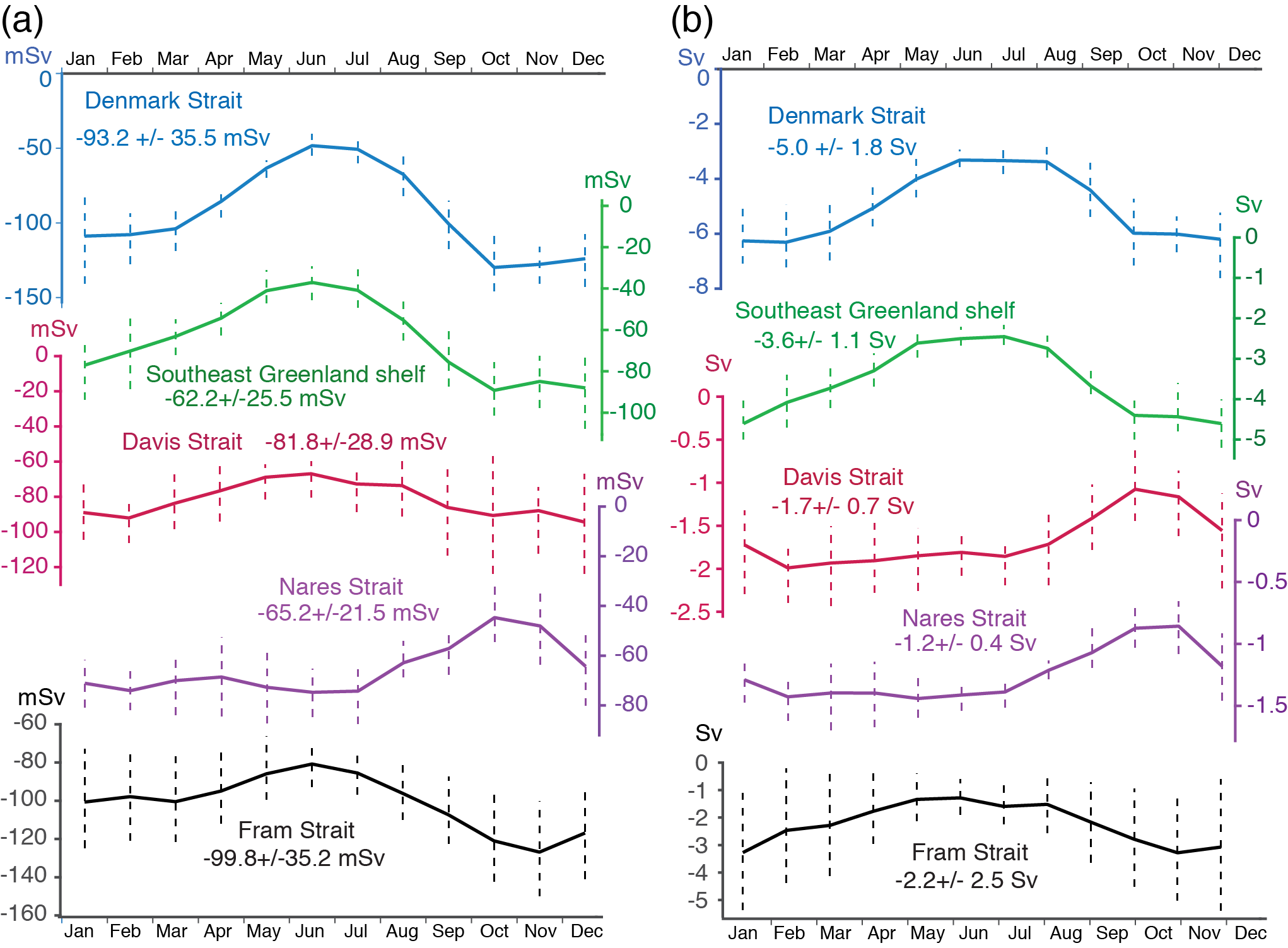


Figure S1. Monthly climatology of freshwater (a) and volume (b) fluxes (mSv and Sv, respectively) from the 0.08 AO HYCOM-CICE experiment “GR+”. The freshwater flux is calculated relative to salinity 34.8. The vertical dashed lines range from the 25^th^ to the 75^th^ percentiles.


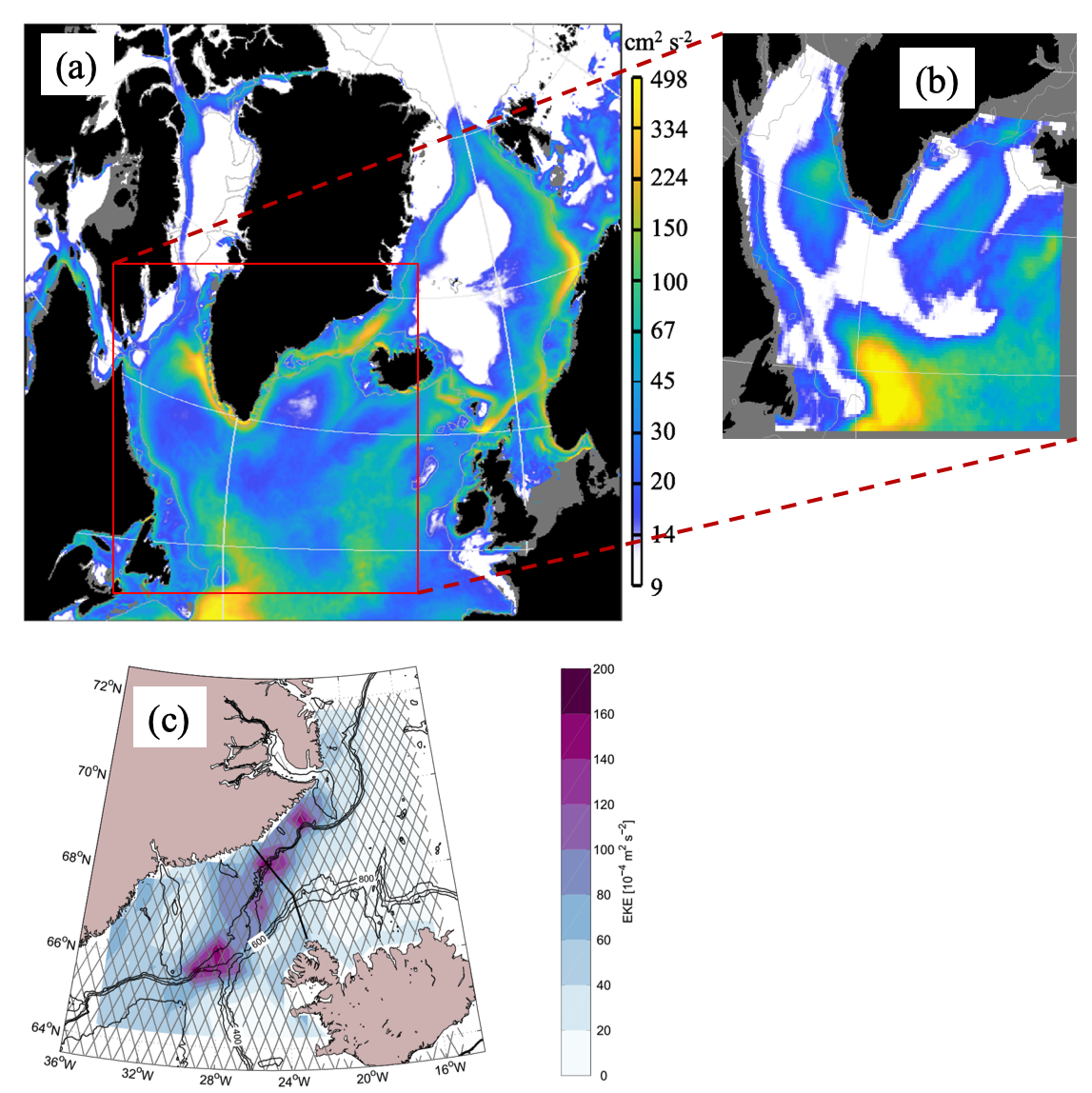


Figure S2. (a) Mean eddy kinetic energy (cm^2^ s^-2^) in the upper 50 m from the 0.08 AO HYCOM-CICE experiment “GR+”. The spatial pattern of the mean EKE has a good general agreement with available estimates derived from drifters and satellite observations showing maximum EKE in the Gulf Stream, near the southern Greenland shelf, and in the eastern Nordic Seas (Richardson, 1983; Heywood et al., 1994; Jakobsen et al., 2003). Note a local maximum of the EKE in the Denmark Strait discussed in (Havik et al., 2017). (b) mean EKE derived from AVISO altimetry. Altimetry-based EKE has higher values near the Grand Banks and Newfoundland Island compared to the model. There is a broader region of elevated EKE in norther Labrador Sea compared to the HYCOM although the model EKE has higher peak values in the region.


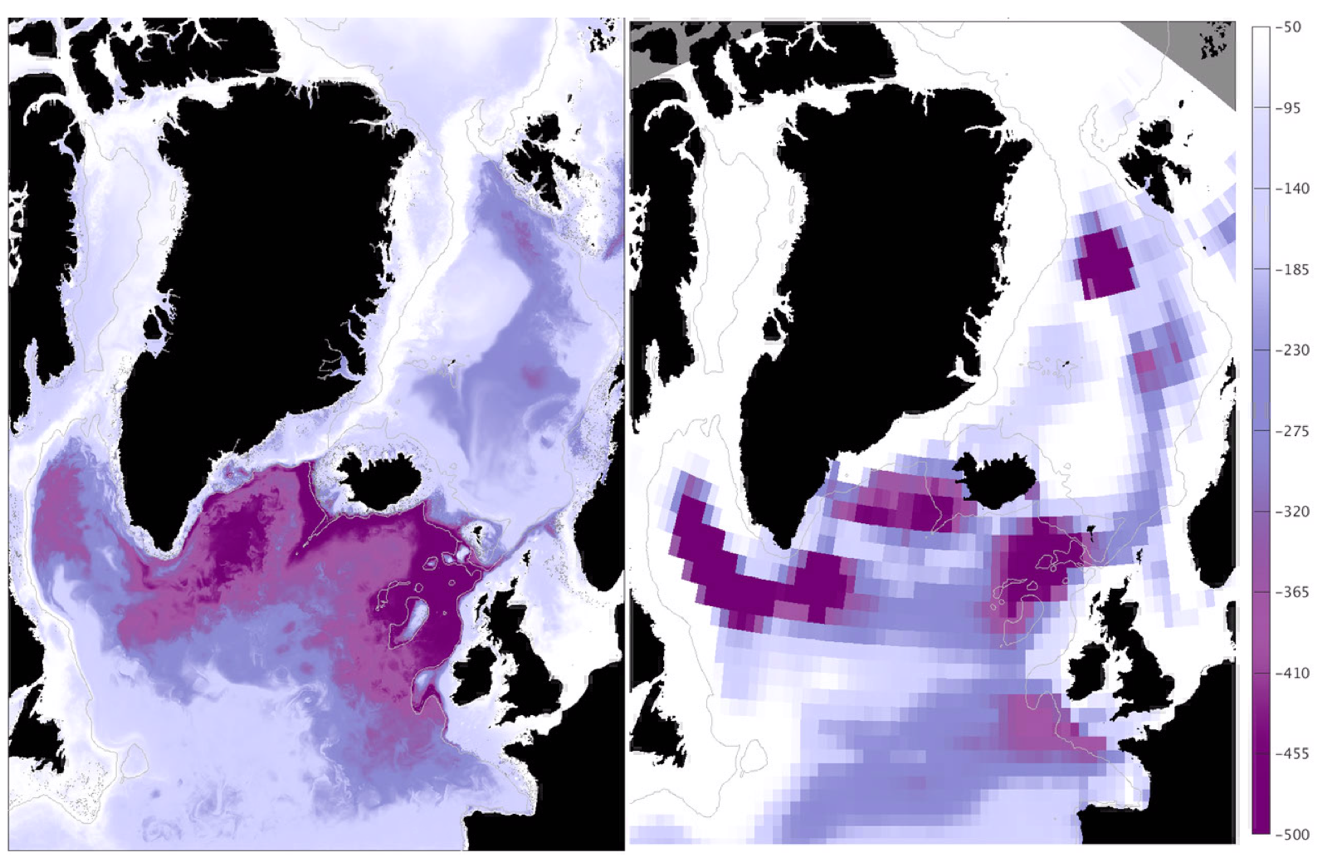


**Figure S3.** February mean mixed layer depth (m) from the 0.08 AO HYCOM-CICE and subsurface temperature and salinity data from the UK Met Office Hadley Center observations data set version EN.4.1.1 (downloaded fromhttp://www.metoffice.gov.uk/hadobs/en4/on 17 November 2015). The fields are averaged over 2010–2016. The mixed layer depth is estimated using a methodology of (Kara et al., 2003). The model simulates deep mixed layer in the Labrador and Irminger Seas, northeastern North Atlantic in agreement with the EN4 data. Deep mixed layer in the central Greenland Sea is not present in the simulations.

Movie S1. Propagation of the passive tracer tracking the Greenland freshwater released at the freshwater sources along the coast of Greenland. The colors depict a fraction of the Greenland tracer accumulated in the model grid cells in the upper 50 m on a natural logarithmic scale. The animation can be accessed here: <https://www.youtube.com/watch?v=0nX-mbrg0Co&t=24s>
